# Does Each Menstrual Cycle Elicit a Distinct Effect on Olfactory and Gustatory Perception?

**DOI:** 10.3390/nu13082509

**Published:** 2021-07-22

**Authors:** Žana Stanić, Ajka Pribisalić, Maria Bošković, Jasna Bućan Cvitanić, Kristina Boban, Gabriela Bašković, Antonija Bartulić, Suzana Demo, Ozren Polašek, Ivana Kolčić

**Affiliations:** 1 Department of Integrative Gynecology, Obstetrics and Minimally Invasive Gynaecologic Surgery, General Hospital Zabok and Hospital of Croatian Veterans, Bračak 8, 49210 Zabok, Croatia; staniczana@yahoo.com; 2 Department of Public Health, University of Split School of Medicine, Šoltanska 2, 21000 Split, Croatia; ajka.pribisalic@mefst.hr (A.P.); opolasek@mefst.hr (O.P.); 3 Department of Immunology and Medical Genetics, University of Split School of Medicine, Šoltanska 2, 21000 Split, Croatia; maria.boskovic@mefst.hr; 4 Department of Anaesthesiology, Reanimatology and Intensive Care, General Hospital Koprivnica, Zeljka Salingera 1, 48000 Koprivnica, Croatia; jasnabucan@gmail.com; 5 Primary Health Centre Zagreb West, Trsje 19b, 10000 Zagreb, Croatia; kristina.boban90@gmail.com; 6 Department of Cardiology, General Hospital Bjelovar, Ul. Antuna Mihanovića 8, 43000 Bjelovar, Croatia; gbaskovic@gmail.com; 7 Department of Anaesthesiology and Intensive Care, University Hospital Split, Spinčićeva 1, 21000 Split, Croatia; antonija.bartulic@gmail.com; 8 Primary Health Center of Split-Dalmatia County, Kavanjinova 2, 21000 Split, Croatia; suzana_balic@hotmail.com

**Keywords:** olfactory perception, gustatory perception, menstrual cycle, oral contraceptives, suprathreshold taste intensity, taste hedonics

## Abstract

The obesity pandemic has brought forth a scientific interest in food intake and sensory perception interactions. Olfactory perception and gustatory perception are very complex and under the influence of many factors, including the menstrual cycle. This study aims to clarify conflicting findings on the influence of the menstrual cycle on olfactory and gustatory perception. Women were assessed during four consecutive phases of one complete cycle (mid-follicular, ovulatory, mid-luteal, and late luteal phases (*N* = 21)), in contrast to women measured across the same phases belonging to two menstrual cycles (*N* = 29). Additional control groups were men (*N* = 17), postmenopausal women (*N* = 14), oral contraceptive users (*N* = 10), and women with an anovulatory cycle (*N* = 8). Olfactory threshold, odor discrimination, and identification were tested using the “Sniffin Sticks“ test kit. Suprathreshold intensity and hedonic ratings for sweet, salty, sour, and bitter solutions were assessed. One-way ANOVA and ANOVA for repeated measurements was applied in the analysis, along with linear and trigonometric data fitting and linear mixed models. Linear increases in olfactory discrimination, identification, and overall olfactory performance were observed only in women followed across a complete menstrual cycle. Compared to other groups, these women displayed a cyclic pattern characterized by a predilection for sweet solution; reduced distaste for salty and sour solutions; and increased intensity perception of salty, sour, and bitter solutions towards the end of the cycle. These results suggest that a distinct hormonal milieu of a complete menstrual cycle may be affecting both olfactory and gustatory perception.

## 1. Introduction

Smell and taste are characterized as chemical senses and are crucial for interactions with the environment. The olfactory system functions as a type of safety alarm system, in charge of detecting dangerous chemicals in the air or in spoiled food. It is also important for nutrition and sexuality—the fundamental pleasures of life [1,2]. In humans, olfaction also impacts mood, encourages mother-to-infant bonding, guides food preferences [3], and affects well-being and the overall quality of life [4]. It is also associated with longevity, while the loss of smell can be an early sign of a neurodegenerative disease, such as Alzheimer’s or Parkinson’s disease [1,4].

Sense of taste or gustation plays a crucial role in food preferences by helping us identify both safe and nutritious foods, as well as energy-dense and nourishing foods [5]. These abilities were of utmost importance for our evolutionary development [6].

Many factors have demonstrated a modifying effect on the perception of taste and odor stimuli. Some of the important determinants include sex [7], age [4,8], genetic factors [9], ethnicity [10], obesity [11], metabolic syndrome [12], diabetes [13], insulin resistance [14], hunger [15], certain medications [16,17], and possibly even the oral microbiota composition [5] and hormonal fluctuations, especially during pregnancy [18] and throughout the menstrual cycle [19]. Gender differences are reported in almost every sensory system in humans [7]. Many studies have shown that women outperform men on tests of odor detection (threshold), identification, discrimination, and memory in tests based on social odors, even for odors that are stereotypically regarded as male [7]. It has been speculated that the better proficiency in odor performance in women is caused by hormonal factors. During sexual maturation, olfactory acuity in women heightens and starts to oscillate across the menstrual cycle [20], leading to a more smell-oriented pattern of behavior in a variety of social and sexual contexts in women compared to men [7]. Moreover, there are marked anatomical sex differences in the human gustatory sensorium at the level of the taste receptors. Women have more fungiform taste papillae than men, and those papillae have more pores leading to the taste buds [21]. Gender differences are further enhanced by observed physiological differences in electrophysiological responses of the gustatory system, which are believed to be connected to both organizing and activating effects of androgens and estrogens [21].

The menstrual cycle and hormonal contraceptives have both shown effects on the intrinsic connectivity of resting-state brain networks associated with emotion processing, olfaction, audition, vision, coordination, and other cognitive functions [22]. Additionally, it was shown that the menstrual cycle has a detectable influence on the human brain structure. Specifically, estradiol has an effect on the gray matter volumes of the bilateral hippocampus during the pre-ovulatory phase, while progesterone has an effect on the increases in the gray matter volumes of the right basal ganglia after ovulation, suggesting possible menstrual-cycle-dependent changes in cognition and emotion [23].

Even though the investigation of the effects of the menstrual cycle and sex hormones on olfactory and gustatory performance started many decades ago, this issue is still far from being entirely resolved. As pointed out in previous reviews, some studies have found strong relationships between menstrual cycle, oral contraceptive use, and pregnancy, effecting smell and taste perception, while others failed to confirm these hypotheses [7,24,25,26]. For instance, several studies have reported that the olfactory threshold varies across the menstrual cycle, with increased sensitivity during the ovulation or luteal phase (decreased threshold) and decreased sensitivity during the menstruation or follicular phase (increased threshold) [19,27,28], although several studies have presented opposing results (for details, see the paper by Derntl et al. [19]). Additionally, distinct differences have been observed for specific odors, indicating the existence of odor specificity brought about by specific reproductive hormones [29].

Understanding whether high-energy food overconsumption and resulting obesity are the results of hormonally induced changes on gustation and food consumption is of particular interest. Electrogustometry measurements performed by Kuga et al. showed a minimal decrease in gustatory threshold during the luteal phase compared to the follicular phase [30]. A lower threshold for sucrose was observed in the preovulatory phase of the menstrual cycle compared to follicular and luteal phases [31]. Sensitivity to sour taste was decreased in the luteal phase [32]. Verma et al. observed cyclic variations in salt preferences across the cycle, with the highest preference for salted popcorn occurring during the luteal phase [33], which is in accordance with the report by Alberti-Fidanza at al. [34].

The limitations of previous studies on sensory perception variations throughout the menstrual cycle have included the involvement of only women of reproductive age, small sample sizes, and measurements being performed only in follicular and luteal phases of the menstrual cycle, in addition to measurements not being performed within the same cycle or with the same women. Such variations in study design might have brought about the discrepancies between the previously published results; hence, the aim of our study was to investigate the distinct effects of all four phases of the complete menstrual cycle on both olfactory and gustatory performance in women. In order to assess the effects of a complete menstrual cycle defined as a unit of observation, we included a control group of women tested across the same phases of the cycle in two consecutive cycles. This provided us with an estimate of the hormonal impacts of cycle phases taken out of the hormonal milieu of one complete menstrual cycle. In order to exclude possible confounders and understand the influence of different sex hormone profiles on sensory perception, we included four additional control groups, namely men, postmenopausal women, oral contraceptives users, and women with an anovulatory cycle.

## 2. Materials and Methods

### 2.1. Participants

A total of 112 adult participants were included in this observational follow-up study. Medical students, nurses, and doctors served as volunteers for the study. All of the participants were blinded to the research question regarding the influence of the menstrual cycle on sensory perception in order to avoid any bias. Participants were told that the principal aim was to investigate the differences in sensory perception between men and women and different age groups. All but one researcher was unaware of a subject’s group allocation (except for cases of obvious characteristics. such as male gender or age in postmenopausal women) and the phase of the menstrual cycle. The unblinded researcher was in charge of recruitment, explained the study protocol and all the procedures to each participant individually, answered all of their questions, and obtained signed informed consent. She further acquired medical and reproductive history information, instructed women on how to perform the home ovulation test, and created an individual plan for each participant regarding their measurement schedule. This researcher did not participate in any of the tests or further data collection and analysis.

**Figure 1:**
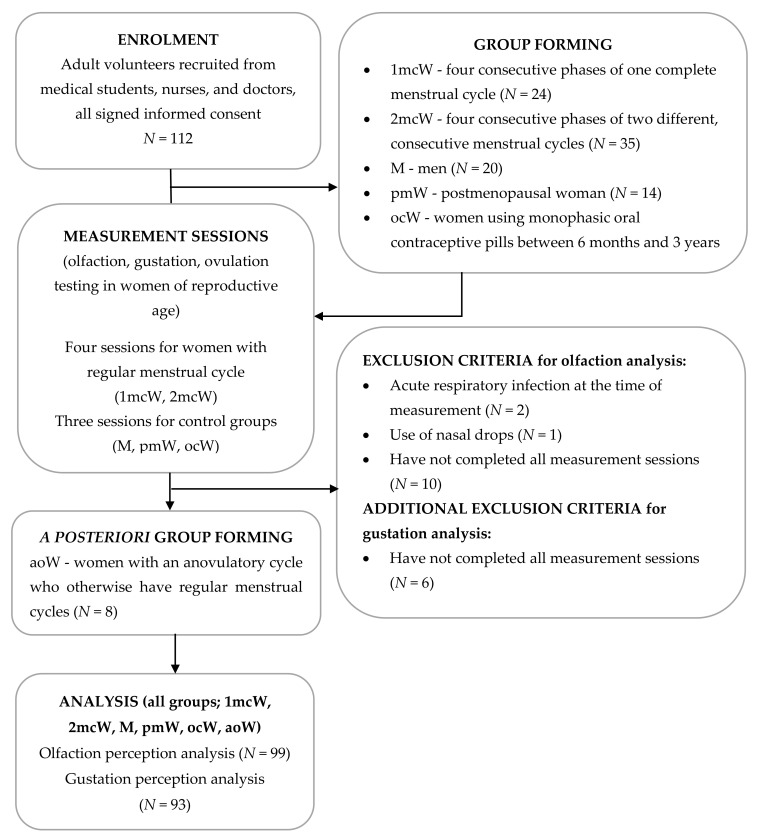
Flowchart of the study. All study groups, exclusion criteria, and numbers of subjects who were lost to follow-up are shown, along with the sample size included in the final analysis of the data (1mcW—women measured across consecutive phases of one complete menstrual cycle; 2mcW—women measured across phases of two different, consecutive menstrual cycles; M—men; pmW—postmenopausal women; ocW—oral contraceptive users; aoW—women with an anovulatory cycle).

Participants were allocated to six groups, one of which was formed *a posteriori* (Figure 1). There were two investigated groups of women with regular menstrual cycles and four measurement tests. The first group was assessed in four consecutive phases of one complete menstrual cycle (1mcW; *N* = 24), starting with the mid-follicular phase, then followed by the ovulatory, mid-luteal, and late luteal phases. The second group of women with regular menstrual cycles was also measured four times, although across phases belonging to two different, consecutive menstrual cycles (2mcW; *N* = 35). The first measurement was taken either in the ovulatory (*N* = 9), mid-luteal (*N* = 20), or late luteal phase (*N* = 6), then each woman subsequently continued from there to be measured an additional three times.

Three control groups were included: men (M; *N* = 20), postmenopausal women (pmW; *N* = 14), and women who had been on monophasic oral contraceptive pills for at least 6 months to 3 years (ocW; *N* = 10). An additional *a posteriori* control group was formed, consisting of women with an anovulatory cycle of regular length, as diagnosed by results of the urinary ovulation test (aoW; *N* = 9) (Figure 1). To avoid the effect of perimenopausal symptoms such as hot flashes, sleep problems, and mood changes, 5 to 10 years had elapsed since the last menstrual bleeding for postmenopausal women included in the study, while women using oral contraceptive pills were tested while on the pill.

The four control groups represent different sex hormone profiles, including non-cyclic and cyclic hormonal changes, covering the effects of male hormones, low levels of estrogen, and progesterone in postmenopausal women, women without cyclic hormonal changes due to oral contraceptives, and women without ovulation and a luteal phase in an anovulatory cycle. All participants from the four control groups were asked for three measurements. Measurements were scheduled in approximation to the women subjected to the four measurements (1mcW and 2mcW groups), with at least 4 and up to a maximum of 15 days between each of the measurement sessions.

Participants were asked not to smoke, brush their teeth, chew gum, or eat or drink anything aside from water at least one hour prior to testing and not to come in hungry or thirsty. They were also advised to refrain from using perfume on the day of the measurement.

All of the participants were in good general health, as assessed by a medical doctor using a medical history questionnaire, including questions about previously established diagnosis and treatment for hypertension, coronary heart disease, cancer, neurosis, depression, diabetes (both types), kidney disease, thyroid disease or dysfunction, anemia, autoimmune disease, asthma, and respiratory allergies. Four menopausal women reported arterial hypertension and five menopausal women reported hypothyroidism medicated by levothyroxine, as well as one woman in the ocW group and one in the 1mcW group. Nobody reported having diabetes, depression, neurosis, or other chronic diseases. We excluded five participants who completed only one measurement session, five participants who completed three instead of four measurement sessions, two participants who had acute respiratory infections (common cold) at the time they were due for a measurement session, and one participant who appeared to be hypoosmotic brought on by the misuse of nasal drops (Figure 1). This yielded a sample size of 99 participants in the olfaction perception analysis (17 men, 14 postmenopausal women, 10 women taking oral contraceptives, 8 women with anovulatory cycles, 21 women measured across one complete menstrual cycle, and 29 women measured across phases of two different, consecutive menstrual cycles). In total, 93 participants were enrolled in the taste perception analysis (16 men, 14 postmenopausal women, 8 women taking oral contraceptives, 8 women with anovulatory cycles, 18 women measured across one complete menstrual cycle, and 29 women measured across phases of two different, consecutive menstrual cycles). All of the participants were free from active respiratory allergies and ENT diagnosis based on self-reported data and assessment through a medical doctor.

### 2.2. Measurements

Each participant filled in the detailed self-administered questionnaire, which consisted of demographic and socioeconomic questions, questions on allergies and acute respiratory infections, dietary habits, food preferences, alcohol intake, and smoking habits. Additionally, the influences of satiety and thirst level, as well as subjective levels of stress, anxiety, and happiness, were noted due to their recognized influence on sensory perception [15] through the application of a visual analogue scale prior to the measurements (1 = not at all hungry/thirsty/stressed/anxious/happy; 10 = extremely hungry/thirsty/stressed/anxious/happy). Participants filled out identical questionnaires during each measurement session. Information on demographic and socioeconomic status was collected only on the initial visit.

Weight, height, hip, and abdomen circumference were measured using standard procedures in the first measurement session only, while olfaction and taste measurements were performed at all follow-up sessions. We calculated the body mass index (BMI), waist-to-hip ratio (WHR), and waist-to-height ratio (WtHR) as measures of nutritional status.

The beginning of each subsequent measurement session was kept within 2 h of the initial testing time for each participant. For example, if a participant started testing at 10 am the first time, the consecutive testing times were somewhere between 9 and 11 am. Each session lasted around 1.5 and 2 h, including answering the questionnaire and conducting all of the measurements. All tests and measurements were performed at the Medical School, University of Split, in an air-conditioned laboratory with the temperature set to 23 degrees Celsius in spring and 24 degrees Celsius in summer, from March through to September 2015. All procedures adhered to the Declaration of Helsinki for Medical Research involving Human Subjects, and two local ethics committees approved the study (University of Split School of Medicine and University Hospital Centre Split). All participants provided written informed consent prior to the study enrolment. The participants did not receive any financial gain for their participation in the study but were informed of their own results with interpretation.

### 2.3. Olfaction Measurement

Olfaction was assessed using the extended “Sniffin Sticks“ test kit (Burghart Instruments, Germany), which includes the threshold, discrimination, and identification tests [35]. Unlike a similar study that used n-butanol, our threshold test utilized 2-phenylethanol (rose smell) [19]. However, the comparison between odor thresholds for 2-phenylethanol and butanol showed a significant correlation (r = 0.60, *p* < 0.001), indicating that 2-phenylethanol can be used as an alternative to n-butanol [36].

The threshold test was always performed first and participants were blindfolded. Pen-like odor-presenting sticks were presented in a staircase method, whereby the 3-alternative forced choice procedure was applied (one pen with 2-phenylethanol and two with only diluent), starting from the highest of 16 dilution steps (less intense smell) and moving towards smaller dilutions (more intense smell). The dilution that was initially recognized two times in a row was considered as a starting point for the rest of the procedure and participants were presented with the next highest dilution level. The threshold test gave a total of seven reversal points, while the threshold score was calculated as the average recognized dilution number for the last four reversals [35].

The discrimination test consisted of 16 triplets, whereby two pens out of each triplet had the same smell and one pen was different. The goal for the blindfolded participant was to correctly identify the different smell, again using the forced choice approach. Discrimination test scores were generated as the sum of correctly identified triplets [35]. The last test performed was the identification test, which consisted of 16 common odorants. Participants (not blindfolded) had to choose the correct odor from a list of four possible answers. The final score was calculated as the sum of the correct answers [35]. The scores obtained in all three olfactory tests were summed up to obtain the overall olfactory performance, giving a TDI score (threshold, discrimination, and identification) [35].

The selection of pens for each triplet in the threshold and discrimination tests was randomized using tables of random numbers. The same selection of pens was applied for the identification test. Randomly numbered tables were prepared prior to testing to avoid predictability of odor presentation in subsequent measurement sessions, especially in the case of the identification test. Each pen was presented only once for about 3 s, 2 centimeters away from both nostrils (birhinally performed test). The examiner was wearing odorless nitrile gloves and was not wearing any perfume or deodorant.

### 2.4. Gustation Measurement

Whole-mouth taste abilities were assessed at the suprathreshold level for four basic tastes, namely sweet, salty, sour, and bitter, using one solution concentration per taste. Taste intensity and hedonics were measured using water solutions of sucrose for sweet (70 mM) [37], sodium chloride for salty (137 mM) [37], L-ascorbic acid for sour (33 mM) [38,39], and quinine HCl for bitter taste (0.18 mM) [40]. In comparison with previous studies, our concentrations of sweet and salty solutions were on the lower end of the previously used tastant concentrations for suprathreshold testing [37], while being similar to sour and bitter concentrations [39,40]. We conducted pilot testing on a subsample (*N* = 10) using both halved and full concentrations of each solution to confirm that all participants would be able to identify the solutions correctly and be able to perceive them at the suprathreshold level, without aversion being provoked by excessively high concentrations.

The water used for the preparation of solutions was of mild taste and originated from natural springs (Jana brand, Jamnica plus company, Zagreb, Croatia; http://www.jana-water.com (accessed on 05 July 2021)). This water contained 64.2 mg/L of Ca^2+^, 32.1 mg/L of Mg^2+^, 1.7 mg/L of Na^+^, and 2.8 mg/L of Cl^-^, considered a low total mineral content [41]. Solutions were prepared daily at room temperature and the bottles were wrapped with aluminum foil to protect the fluids from exposure to light and subsequent degradation, especially the one containing ascorbic acid.

The perceived taste intensity was measured using a labeled magnitude scale (LMS) [42]. Verbal labels were placed along the vertical line without numbers written next to the labels (“no sensation” was placed at 0 mm, “barely detectable” at 2 mm, “weak” at 7 mm, “moderate” at 20 mm, “strong” at 40 mm, “very strong” at 61 mm, and “strongest imaginable” placed at 114 mm from the beginning), as in previous studies [43]. Since this kind of scale may not be intuitive, the administrator demonstrated the usage of the scale. The participants were instructed to place their mark of perceived intensity for the tested solution anywhere on the line, regardless of the placing of the expressions, while comparing the presented stimulus against the strongest one they recall experiencing [42].

The solution affect (hedonic rating) was measured using a labeled affective magnitude scale (LAM), with a total line length of 100 mm, where semantic labels “greatest imaginable dislike”, “neither like nor dislike”, and ”greatest imaginable like” were placed at 0, 50, and 100 mm, respectively [44]. Labels “like extremely”, “like very much”, “like moderately”, “like slightly”, “dislike slightly”, “dislike moderately”, “dislike very much”, and “dislike extremely” were placed along the line as suggested by Schutz at al. [44]. The administrator measured the distances between 0 mm and the mark each subject made on the scale and recorded these in millimeters for each tastant. To indicate the negative effects of the scale, the whole scale was turned into a range from −50 to +50 mm by subtracting the number 50 from the actual values, whereby −50 means “greatest imaginable dislike”, 0 means “neither like nor dislike”, and +50 mm indicates ”greatest imaginable like”.

The presentation of the solutions was random, except for bitter taste, which was always presented last. Participants received a disposable cup with 10 mL of each tasting solution kept at room temperature, without knowing which solution was presented to them. Participants were instructed to swish or rinse the fluid in their mouth for a few seconds before drinking to be better able to perceive the taste and intensity. Between each tasting, there was a break of at least one minute with a mouth rinse with the same type of water used for the preparation of the solutions.

### 2.5. Ovulation Determination

All women of reproductive age had regular menstrual cycles, with cycle lengths of between 22 and 35 days (average 28.7 ± 2.6 days), based on the previous 6 cycles. Ovulation was determined using a self-administered urinary test at home (One Step LH ovulation test with high sensitivity of 20 mIU/mL, FDA-approved, ISO 134852003-certified, produced by AI DE Diagnostic Co., Ltd., P.R. China). For example, it was shown that urinary ovulation tests with a threshold of 25–30 mIU/mL yielded the best predictive value for ovulation within 24 h [45]. The test provides visual results in 2–5 min, determining sharp increases in luteinizing hormone (LH) concentrations in urine—the so-called “LH surge”—which precedes ovulation. Ovulation is usually expected within 24 h of the observed LH surge. The right time to test for the LH surge was calculated individually, based on information on the duration of the last six menstrual cycles, and was initiated at least 3 days before anticipated ovulation. The same information on the self-reported average length of the menstrual cycle was used to determine the timing of mid-follicular, mid-luteal, and late luteal measurement sessions in women with regular cycles.

Women repeated ovulation tests for at least 10 consecutive days before conclusion of an anovulatory cycle was considered. No distinct gynecologic reasons for anovulatory cycles, for example polycystic ovarian syndrome, were detected by gynecologists in any of these women. Instruction leaflets were provided to every woman, along with a thorough explanation of the testing preparation and procedure and interpretation of results.

All participants tested with urinary ovulation tests were advised to report positive results of the ovulation test immediately so the ovulatory sensory testing session could be arranged within the next 24 h. Participants who failed to register ovulation were classified into a control group of women with anovulatory cycles (aoW) and their sensory perception was tested one more time.

### 2.6. Statistical Analysis

Mean and standard deviation were calculated for continuous variables with normal distribution (tested with the Kolmogorov–Smirnov test), while the median with the interquartile range (IQR) was calculated for ordinal variables and continuous variables with non-normal distribution). Categorical variables were described using absolute numbers and percentages. Differences between the six groups were tested using Fisher’s exact test for categorical variables, while for numerical variables we used one-way ANOVA with Tukey’s HSD post hoc test or the Kruskal–Wallis test with Mann–Whitney U test as a post hoc test (and for ordinal variables), depending on the distribution.

The differences within each of the six groups included in the study were tested with ANOVA for repeated measures (for taste and olfactory perception), or its non-parametric alternative, the Friedman test, was performed with Wilcoxon signed ranks test as the post hoc test (for stress, anxiety, happiness, satiety, and thirst levels). If the criteria of sphericity for repeated measures in the ANOVA test was not met, the violation of sphericity was resolved with the Greenhouse–Geisser correction of *p*-values, while post hoc analyses employed a Bonferroni correction.

Additionally, in order to detect the possible existence of linear or cyclic patterns for both olfactory and gustatory perception data across measurement sessions for each group separately, we applied the linear and trigonometric fit method using a web-based service available at https://www.mycurvefit.com (accessed on 05 January 2021). The existence of the linear fit was tested using the formula:

(1) Y=mx + c

where m represents the slope and c represents the intercept. The formula for the trigonometric fit was:

(2) $$y\;=\;a*\sin\left(x\right)+\;b*\cos\left(x\right)+\;c$$

where a and b represent amplitudes and c represents the vertical shift. The fitting parameters were based on group averages across the measurement sessions. The linear fit process was performed for all groups, while the trigonometric fitting was limited to 1mcW and 2mcW groups, since trigonometric fitting requires at least four data points for computation.

Finally, a linear mixed model analysis was performed in order to replicate the results from ANOVA for repeated measures, while taking into account possible confounding effects of age (continuous variable), smoking (yes/no), and waist-to-height ratio (elevated if ≥0.5).

We computed the achieved power (post hoc power analysis) using the G*Power tool v. 3.1.9.7 [46], reaching a value of 83.6% for analysis of sensory performance in 1mcW and 2mcW study groups, which are the two main groups in this study corresponding to our main hypothesis. Power calculation was performed with the following assumptions: effect size of 0.13 (this was the lowest obtained effect size in our analysis for both smell and taste perception), probability of type I error 0.05, total sample size of 47, with two tested groups and 4 repeated measurements, with a correlation between repeated measurements of at least 0.74, without non-sphericity correction; ANOVA was applied for repeated measurements.

Statistical analyses were performed using IBM SPSS Statistics package v22 (IBM, Armonk, NY, USA). The significance was set at *p* < 0.05.

## 3. Results

### 3.1. Participants’ Characteristics

This study included 99 participants, classified into six study groups. Apart from the postmenopausal (pmW) women being older than all other groups (all *p* < 0.001), no statistical difference between groups regarding age, socioeconomic status, or smoking prevalence was detected (Table 1). Men (M group) and postmenopausal women (pmW) had higher BMIs and waist-to-hip ratio indices compared to the other groups (Table 1).

There were no differences in hunger or thirst ratings between groups for any of the measurement sessions or cycle phases (Supplementary Table S1). Comparisons of repeated measurements of hunger and thirst ratings within each of the groups showed only one significant difference—within the 2mcW group for thirst rating (*p* = 0.037; Supplementary Table S1). There were no differences in perceived stress level (all *p* > 0.177), anxiety level (all *p* > 0.125), or perceived happiness (all *p* > 0.123) for repeated measurements within each of the study groups across measurement sessions (Supplementary Table S1). There were no differences in any of the examined emotional state ratings between women measured during four consecutive phases of one complete menstrual cycle (1mcW) and women measured in four phases across two different and consecutive menstrual cycles (2mcW) (Supplementary Table S1).

### 3.2. Olfaction Perception

#### 3.2.1. Olfactory Threshold

There were no differences in odor threshold for 2-phenylethanol between groups in any of the measurement sessions, and men performed equal to women (Table 2). There was a significant difference in repeated measurements only within the group of postmenopausal women (F(2,26) = 5.84, partial ɳ^2^ = 0.31, *p* = 0.008), with the best sensory performance during the third measurement session, but without a linear increase across measurement sessions (Table 2). This result was also confirmed in the linear mixed model, with significant group–time interactions for the first (*p* = 0.010) and second (*p* = 0.022) measurements compared to the third measurement in postmenopausal women (Table 3). A significant linear reduction in odor threshold sensitivity was recorded across the time points in the anovulatory group of women (aoW; *p* = 0.017) and in women across four phases belonging to two menstrual cycles (2mcW group *p* = 0.038; the data presentation for this group follows the cycle phase ordering instead of the timeline of measurement sessions; Table 2). These findings were not confirmed in the linear mixed model, but there was a significant group–time interaction in olfactory threshold in women followed across one menstrual cycle for the 1st measurement (follicular phase) compared to the late luteal phase measurement (*p* = 0.047) (Table 3).

#### 3.2.2. Olfactory Discrimination

Odor discrimination analysis revealed significant differences only within the group of women followed through a single menstrual cycle (1mcW; F(3,60) = 3.872, partial ɳ^2^ = 0.16, *p* = 0.013, Table 2). There was a steady increase of the mean discrimination score across the measurement timeline, starting with the mean of 12.05 ± 2.25 in the mid-follicular phase compared to 13.52 ± 1.25 in the late luteal phase, but without a significant post hoc result (when more stringent Bonferroni correction was applied; Table 2); however, a linear curve fit showed a significant increase in odor discrimination across cycle phases only within this group of participants (*p* = 0.004; Table 2). This was also confirmed in the linear mixed model, where a significant group–time interaction was recorded in women followed across one menstrual cycle (for mid-follicular (*p* = 0.038) and ovulatory (*p* = 0.024) measurements compared to late luteal measurement) (Table 3).

Regarding the between-group comparison of odor discrimination, only the group of postmenopausal women (pmW) showed significantly lower odor discrimination scores in 2nd and 3rd measurement sessions compared to the other five groups (Table 2), while age was confirmed to be negatively associated with odor discrimination ability in the linear mixed model (*p* = 0.046, Table 3).

#### 3.2.3. Olfactory Identification

Odor identification scores in repeated measurements significantly increased only within the 1mcW group (F(3,60) = 8.20, partial ɳ^2^ = 0.29, *p* < 0.001) and ocW group (*p* = 0.036), indicating an improvement in performance (Table 2). After post hoc analyses, the significant difference remained only for the 1mcW group. Namely, there was a statistically significant increase in the late luteal phase measurement (4th measurement, mean of 14.19 ± 1.50) compared to the mid-follicular phase (1st measurement, 12.67 ± 1.98; *p* = 0.001) and ovulatory phase measurements (2^nd^ measurement, 13.38 ± 1.66; *p* = 0.028, Table 2). Additionally, the linear curve fit was significant only in the 1mcW group (*p* = 0.026), and a significant result was also obtained for the group–time interaction in the linear mixed model for this subgroup, as well as for women taking oral contraceptives (Table 3).

#### 3.2.4. Overall Olfactory Performance—TDI Score (Threshold, Discrimination, and Identification)

**Figure 2:**
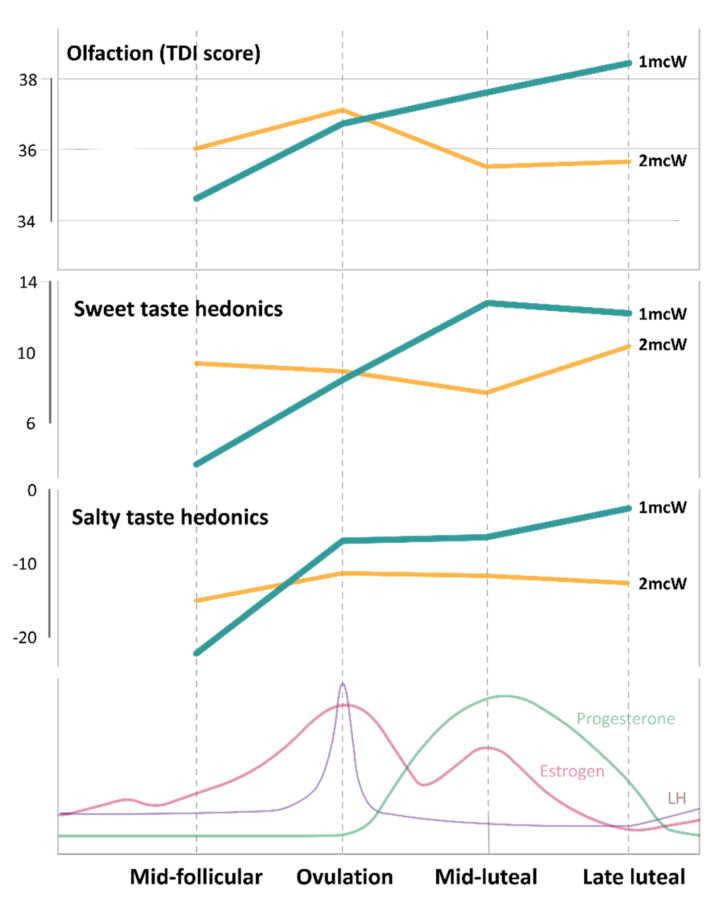
TDI score, sweet taste hedonics, and salty taste hedonics across four phases of the menstrual cycle in two main study groups (1mcW - women measured across consecutive phases of one complete menstrual cycle; 2mcW - women measured across phases of two different, consecutive menstrual cycles; all values are presented as means, while error bars are not shown to improve figure clarity).

Women taking oral contraceptives (ocW group) and women followed across one menstrual cycle (1mcW group) displayed steadily increasing patterns in overall olfactory performance expressed as TDI scores, but only the 1mcW group showed a statistically significant increase (F(3,60) = 7.72, partial ɳ^2^ = 0.28, *p* < 0.001, Table 2). The linear curve fit for the TDI score was again significant only in this group of participants (*p* = 0.039), the same as for the result in the linear mixed model, where the group–time interaction was also significant in this subgroup (Table 3). TDI scores across phases of menstrual cycle in the 1mcW group and 2mcW group are shown in Figure 2.

Men demonstrated differences between the mean TDI score obtained in the first measurement session (mean of 34.85 ± 3.36) and the second one (37.29 ± 3.21; post hoc *p* = 0.028) (Table 2). All groups of younger participants outperformed postmenopausal women (pmW), except during the first measurement session, where men and the 1mcW group did not differ from the pmW group. Men did not show any significant olfactory inferiority compared to women of similar age.

### 3.3. Gustatory Perception

#### 3.3.1. Sweet Taste

Men displayed the highest average intensity rating for the sweet solution during the first measurement (mean of 63.69 ± 26.79), which differed significantly from women in pmW (38.50 ± 26.47) and ocW (26.38 ± 26.75) groups (Table 4). Sweet intensity ratings increased within the group of women taking oral contraceptives over three measurement sessions (ocW; F(2,14) = 8.87, partial ɳ^2^ = 0.56, *p* = 0.003) (Table 4). The linear mixed model also revealed a significant group–time interaction in women taking oral contraceptives and in women followed across one menstrual cycle (Table 5). Additionally, non-smokers had rated the sweet solution as more intense compared to smokers (*p* = 0.013), while participants with an elevated waist-to-height ratio rated the sweet solution as less intense in taste (*p* = 0.014) (Table 5).

Hedonic ratings of sweet solutions did not differ within any of the groups or between groups, except for in the third measurement session (F(5,87) = 3.55, partial ɳ^2^ = 0.17, *p* = 0.006), where men stating liking the solution more (24.25 ± 13.13) compared to postmenopausal women (0.86 ± 19.37) and women followed through two menstrual cycles (8.04 ± 18.78) (Table 4). The linear mixed model also identified men as having a higher hedonic rating of sucrose (Table 6); however, women followed through four phases in one menstrual cycle (1mcW) demonstrated a cyclic pattern of sucrose hedonic rating (trigonometric fit *p* = 0.011), unlike the 2mcW group (Figure 2), while a linearly increasing trend of sucrose propensity was observed in women with anovulatory cycles (*p* = 0.035) (Table 4).

#### 3.3.2. Salty Taste

The only group of participants that showed a significant within-group difference between measurement sessions for salty solution intensity rating was the 1mcW group (F(3,51) = 5.20, partial ɳ^2^ = 0.23, *p* = 0.003). The variation resembled a cyclic pattern with a significant trigonometric fit (*p* = 0.033) (Table 4). The linear mixed model identified a marginally insignificant result for the 1mcW group–time interaction (*p* = 0.052; Table 5).

A similar result was recorded for the salty solution hedonic rating, with a significant within-group difference only in the 1mcW group (F(1.86,31.55) = 9.02, partial ɳ^2^ = 0.35, *p* = 0.001) (Table 4). These women exhibited the strongest level of dislike in the mid-follicular phase (mean of −22.56 ± 21.51), along with reductions of the perceived dislike in the following sessions (−3.17 ± 25.07 in late luteal phase; Figure 2), but without a trigonometric or linear trend (Table 4). These results were also confirmed in the linear mixed model (Table 6). Interestingly, only men displayed a marginally significant linear trend (*p* = 0.042), with a decrease of dislike of salty solution towards the third measurement (F(1.35,20.33) = 5.57, partial ɳ^2^ = 0.27, *p* = 0.020; Table 4), which was also found to be significant in the linear mixed model (*p* = 0.007; Table 6).

#### 3.3.3. Sour Taste

Analysis of the data regarding the within-group difference in sour solution intensity ratings again yielded a significant result only for the 1mcW group (F(3,51) = 3.00, partial ɳ^2^ = 0.15, *p* = 0.039), with a post hoc marginally significant difference between mid-follicular (62.11 ± 26.43) and mid-luteal phase (74.39 ± 24.57; *p* = 0.048) measurements, but without a significant linear or trigonometric fit (Table 4). The linearly increasing trend for the sour solution intensity rating was significant only in the group taking oral contraceptives (ocW; *p* = 0.029), which was also confirmed in the linear mixed model (Table 5) and was marginally insignificant in the anovulatory cycle group (aoW; *p* = 0.052, Table 4).

Sour solution hedonic ratings showed a significant linear trend of decreased dislike towards a slight liking of the solution only in the 1mcW group (−3.06 ± 29.97 in mid-follicular vs. 5.83 ± 24.16 in late luteal cycle phase; *p* = 0.006) (Table 4), but without confirmation of the result in the linear mixed model.

#### 3.3.4. Bitter Taste

There were no significant differences for the ratings of intensity or hedonics of the bitter solution in either the within-group or between-group bivariate analysis (Table 4). The only significant result was within the 2mcW group for the hedonic rating (F(1.94,54.37) = 3.99, partial ɳ^2^ = 0.13, *p* = 0.025), but with no significant post hoc results (Table 4). However, the 1mcW group revealed a significant linear trend for the bitter solution intensity rating, which increased across measurement sessions (starting from 67.50 ± 35.94 in mid-follicular to 75.22 ± 28.23 in late luteal phase; *p* = 0.016) (Table 4). The linear mixed model identified significant group–time interactions for the bitter taste intensity rating in women taking oral contraceptives (Table 5), and for the bitter taste hedonic rating in postmenopausal women, women taking oral contraceptives, as well as women with an anovulatory cycle (Table 6).

### 3.4. Assessment of the Learning Curve Effect

We performed a statistical analysis with the hypothesis that there would be no difference in the learning effect, meaning there would be no better sensory performance in any of the studied groups. The learning effect was indicated as the number of statistically significant results per group across all testing sessions (within-group comparisons for a total of twelve olfactory and gustatory measurements or tests). We compared the 1mcW group with all other groups combined (controls). We detected nine significant results in the 1mcW group (9 out of 12 tests) and ten significant results in all control groups (10 out of 60 positive tests; 5 groups, each had 12 separate tests). Fisher’s exact test yielded a *p* value of 0.0001494, indicating that the frequency of significant results found in sensory performance across measurement sessions in the 1mcW group was significantly more common than in control groups.

## 4. Discussion

The aim of this study was to investigate the influence of the menstrual cycle on both olfactory and gustatory performance. Particularly, we aimed to show the effects of a single, complete menstrual cycle in contrast to the effects of a particular cycle phase as an independent observational unit on sensory perception. Our results demonstrated increases in olfactory discrimination, identification, and overall olfactory performance and distinct changes in gustatory perception only in women followed across one complete menstrual cycle. Furthermore, these results were not replicated in women who were followed through the same cycle phases belonging to two consecutive cycles. Such findings may be of great methodological importance in future studies on the association between sensory perception and food preferences and intake.

Most previous studies on olfactory performance tested only the smell threshold and showed improved performance (i.e., lower threshold) for at least some substances around the time of ovulation or in the mid-luteal cycle phase [19,20,27,47,48]. On the contrary, some studies of smaller sample size found no effect of the cycle phase on olfactory threshold [49,50], or even showed several peaks in olfactory sensitivity during the cycle [51]. We observed a significant group–time interaction in the linear mixed model for odor threshold in women followed across one menstrual cycle, with reduced performance in the mid-follicular phase compared to the late luteal phase. The same increase in performance across one menstrual cycle was detected, but not discussed in detail, in one of the previous studies [19]. Only women who started measurements in the follicular phase presented with an increased threshold sensitivity for n-butanol in the second measurement, which corresponded to the luteal phase in that study [19]. Another subgroup of women included in the same study, followed throughout two menstrual cycles (they started their measurements in the luteal phase, while the second session was conducted in the follicular phase of the next cycle), did not display this outcome [19]. Identical results were obtained in our study. We even detected a linear decrease in threshold sensitivity across the cycle phases within the group of women tested throughout four phases belonging to two consecutive cycles. The same pattern was observed in women with anovulatory cycles. Occasional anovulatory cycles are normal in otherwise regularly ovulating women and they are a complex and heterogeneous phenomenon, resulting from various forms of hypothalamic dysfunctions, hyperprolactinemia, excessive LH and androgen concentrations, lack of progesterone, or constantly elevated estrogen levels [52]; hence, the hormonal profile across an anovulatory cycle can be substantially different compared to the ovulatory cycle. For instance, lower reproductive hormone concentrations were found during the anovulatory cycle, even with repercussions for the next cycle, since women with one anovulatory cycle tended to have lower estradiol, progesterone, and LH peak levels during their next ovulatory cycle [53]. In our study, besides a decrease in olfactory threshold across the measurement sessions, women with an anovulatory cycle had an increase in sweet solution hedonic rating and a borderline insignificant increase in sour solution intensity ratings. To the best of our knowledge, this is the first study to report olfactory and gustatory perceptions across an anovulatory cycle in women who otherwise have a regular menstrual cycle, making us unable to compare our results to previous ones and to interpret the relevance of these findings. Certainly, further studies are needed to elucidate the role of an anovulatory cycle on both sensory function and food intake in humans.

The results of this study point towards the existence of a distinct effect of one complete menstrual cycle on suprathreshold olfactory performance. This statement relies upon an observed increase of odor discrimination and identification scores only within the group of regularly ovulating women followed from the mid-follicular until the late luteal phase of one complete menstrual cycle, while this pattern was absent in the group of women tested across phases in two menstrual cycles. The same result was observed for overall olfactory performance, expressed as a TDI score. These results once more confirm previous findings, whereby increases in identification and TDI scores were detected in the luteal phase compared to the follicular phase only in women followed from the follicular to the luteal phase, and not the other way around [19]. Another study including 17 women detected an increase in TDI score in the mid-luteal phase in comparison to the late follicular or ovulation phase, unlike for the threshold, discrimination, and identification scores [54]; however, this study did not explicitly describe the order of the three measurements and whether women were followed across one complete cycle or across two or more cycles [54].

Our data and previous study results, which indicated an increase in olfactory performance, correlate well with the increasing concentrations of estrogen and progesterone across the menstrual cycle. For a long time it was suspected that menstrual cycle oscillatory hormone levels had an array of physiological effects, and only lately have scientists managed to unearth the evidence for their effects on virtually every organ system in the body [55]. The fluctuations of sex hormones have a role in various brain functions, especially in domains of cognition, emotion, sensory processing, and appetite [55]. Sex hormone receptors were found in several nuclei associated with central gustatory pathways, pointing to the possibility of modulation of central taste processing, together with estrogen modification of taste-elicited activity in the periphery [25]. For instance, it was found that taste receptor cells present a direct target for estrogen in both the nuclear and plasma membrane forms of the estrogen receptor in mouse taste cells [56]. Three estrogen receptors (ERs) have been discovered so far; Erα, ERβ, and G-protein coupled ER1 (GPER1). ERs are found in multiple brain regions, from the most rostral regions of the forebrain to the cerebellum, and high levels of GPER1 were observed in the olfactory bulb, providing evidence for the mechanism of estrogen’s effect on olfactory perception [57]. Acting through the nuclear ERs to elicit genomic effects to regulate the transcription of proteins suggests that estrogens alter the production of multiple proteins in the central nervous system, including growth factors, cytokines, and apoptotic factors, while action in the cell membrane induces rapid non-genomic effects, such as altering membrane permeability and activating second messenger cascades [57].

Previous studies have shown that taste perception, food preferences, and food cravings in women are not uniform across the menstrual cycle [21,31,58]. The majority of these studies investigated only the association between sweet tastant sensitivity perception and hedonic ratings with sweet food intake, preferences, and cravings for sweet foods, with some of them not taking into account the menstrual cycle effect [59]. A recent scoping review found as many as twelve distinct categories of sweet taste determinants and a considerable amount of methodological variability, with mixed results regarding reproductive hormone effects on sweetness preference [60]. Our study showed increased intensity rating of sweet taste of sucrose in women using oral contraceptives and a similar increase in women across phases in a single menstrual cycle, peaking during the mid-luteal phase. The majority of previous studies examined only the threshold sensitivity, detecting an increase in sweet taste sensitivity in parallel with an increase of estradiol in the periovulatory phase or during the luteal phase [31,34,61]. Studies investigating suprathreshold sweet taste intensity and hedonics across the menstrual cycle phases are scarce in the literature [62], preventing further comparison of our results with previous findings, especially since threshold and suprathreshold intensity ratings for sweet taste were shown not to be well correlated [37]. Nevertheless, our results may provide a missing link between sex hormones oscillation across the menstrual cycle and increased food intake. Indeed, increases in appetite, increased craving for chocolate and other sweets, as well as craving for salty flavor, and total craving score were observed during the late luteal phase as compared to the menstrual, follicular, and ovulatory phases [58]. Furthermore, it was reported that sweet hedonic liking correlated positively with total energy and carbohydrate intake [59]; however, elucidation of the association between sweet preference and obesity remains challenging. For instance, a recent study showed that the effects of sweet propensity patterns on anthropometric characteristics depended on age, which might reflect different levels of exposure to the obesogenic environment, while the fat-free mass was most strongly associated with liking sweet foods, unlike BMI or body fat [63].

Ovarian hormones are also important regulators of blood pressure and water regulation systems [64], and they may contribute to the perceived fluid retention across the menstrual cycle [65]. Salty taste perception change across a menstrual cycle could be one of the factors behind this phenomenon, or these two processes may simply be affected by another common mechanism. Previous studies have shown changes in salty taste threshold across the menstrual cycle [61], as well as salt preference [66]. Contrary to these findings, another study failed to find differences in either preference ratings or ratings of saltiness of samples during the menstrual cycle [67]. Our study identified an increase in the salty solution intensity rating, being on average the lowest in mid-follicular phase and peaking in the mid-luteal phase in women followed across a single menstrual cycle. No such indication of cyclicity was detected in women followed across phases belonging to two menstrual cycles. Hedonic ratings of salty solutions revealed that women tested across phases of a single cycle perceived this solution most negatively in the mid-follicular phase, especially compared to the late luteal phase, when the least negative ratings were observed. A similar finding was recorded in women rating popcorn sprayed with five different concentrations of salt solutions, where women in the luteal phase preferred unsalted popcorn significantly less than women in ovulatory or follicular phases [66]. Another study using a similar design showed that the preference for salted popcorn was highest during the luteal phase, while unsalted popcorn was preferred in the menstrual phase [33]. These observations and our findings of increased intensity perception and hedonic valence of salt across the menstrual cycle could be of real importance and clinical value, due to the high consumption of salt and high prevalence of hypertension and heart disease in modern societies.

Sour solution ratings were similar to the ratings for the salty solution in women followed across a single menstrual cycle, with a linear decrease of dislike towards the end of the cycle coupled with an increase in intensity perception and significantly higher ratings in the mid-luteal phase than in the mid-follicular phase. The literature provides us with very few studies regarding the effects of the menstrual cycle on sour taste perception. One previous study found a lower threshold sensitivity to sour taste during the luteal phase of the menstrual cycle compared to the follicular phase, while they found no difference for salty, sweet, or bitter taste [32]. The authors also identified a negative association between sour taste perception and ghrelin concentration in the luteal phase [32]. Ghrelin is a “hunger” hormone, which increases appetite and may affect food intake. Interestingly, ghrelin was found within the taste buds of the tongue in mice, and ghrelin receptor knockout male mice exhibited significantly reduced taste responsivity to sour and salty tastants [68]; however, the actual effects of these findings remain to be explained in further studies.

Bitter taste perception followed a linear pattern of increased intensity ratings only in women followed across a single menstrual cycle, even though women taking oral contraceptives and those with an anovulatory cycle also demonstrated increases in perceived intensity of bitter solution along the measurement sessions, although the results were statistically insignificant. These findings are in line with previous results showing an increase in threshold sensitivity to bitter taste with an increase of progesterone [34], which happens after ovulation in the second half of the menstrual cycle. The effects of progesterone on bitter taste perception were also investigated during pregnancy (due to a steady increase of progesterone concentration). Interestingly, very low consistency between studies was observed, making bitter taste the taste modality with the least consensus in pregnancy [18]. Regardless, significant positive associations between bitter taste perception and energy, carbohydrates, and lipid intake were observed in the luteal phase [32]. This is again important from a public health perspective, as this can possibly affect food intake, resulting in increased intake of energy-dense foods and a higher risk of weight gain [5].

The gustatory performance in women on oral contraceptives revealed linear increases in sweet, salty (both borderline insignificant), and sour solution intensity ratings (significant results also in the linear mixed model), while hedonic perception changed only for the bitter taste. Similar results of no differences in hedonic ratings of sweet and savory foods between luteal and follicular phases were obtained in a previous study involving women on oral contraceptives [69].

We included both smell and taste perceptions in our study in order to investigate whether these chemical senses display similar changes due to sex hormone fluctuations across the menstrual cycle. It is important to mention that recent studies in both animals and humans have shown an overlap in the representation of primary tastes and odorants in the brain. For instance, some of the neurons were found to respond to just one of the four primary taste stimuli, while other neurons responded to more stimuli, including to all four [70]. In fact, convergence and interactions between taste and olfactory stimuli are considered to be crucial for flavor processing and perception, and several human brain regions have been identified as being activated by unimodal taste and smell stimuli, although interactions between the olfactory and gustatory inputs have also been found in lateral anterior parts of the orbitofrontal cortex [71]. Additionally, processing of the sensory cues associated with food consumption involves an “interacting network of primary sensory areas” [72]. Multisensory integration that occurs in response to food intake, including taste, smell, texture, temperature, and auditory and visual inputs, results in flavor perception, which is represented at multiple levels of the central gustatory system [73].

Many previous studies have implied the superiority of women over men in olfactory and gustatory capabilities [7,25]. On the contrary, we did not observe any differences in olfactory performance between men and women of generative age. The same result of no gender differences in odor threshold for n-butanol and overall TDI score was reported by Derntl et al., although they did report better odor identification and odor discrimination in women compared to men [19]. These findings are in accordance with the previous results of no observed gender differences in odor sensitivity, although better performance was observed for women for tasks involving verbal processing, such as in odor identification [74]. One of the possible explanations for our finding of no obvious gender differences was selection bias—the majority of men included in this study were medical students who are systematically trained to be proficient in observing and lateral thinking, as needed for their future profession.

Aging has a detrimental effect on sensory perception [35,75]. We confirmed this statement for olfactory perception, showing that postmenopausal women had the lowest scores in discrimination and identification odor tests, as well as for overall olfactory performance (TDI score). Regardless, the differences were not as striking (or it was absent for olfactory threshold) because the average age of women in this group was 56.5 years, while decreased olfactory performance was shown to be most pronounced in people older than 60 years [35]. In addition, the gustatory suprathreshold rating in the group of postmenopausal women was apparently not much worse than in women of reproductive age.

Although there were no statistically significant differences in smoking prevalence between the study groups, we must regretfully point to the high prevalence of smokers in this study. All of our participants volunteered, and to maintain adequate sample sizes within study groups, we decided not to exclude smokers. Additionally, we informed all of the participants about the necessity to restrain from smoking at least one hour prior to the measurement sessions. The analysis of repeated measurements was done within each study group separately; therefore, each study group acted as its own control. Moreover, the only outcome significantly associated with smoking in linear mixed models was sweet taste intensity rating, where non-smokers rated sweet solutions as more intense. Furthermore, the effects of smoking on olfactory and gustatory perception remain contradictory. Some studies have failed to identify significant effects of smoking on either smell or taste impairment [76,77], while others have pointed to an increased risk of olfactory dysfunction in current smokers but not former smokers [78] and to impaired thresholds for sweet, salty, sour, and bitter tastes [79].

There are several limitations to this study. Firstly, small sample sizes were available in some of the study groups, with as little as eight participants in the group of women using oral contraceptives, the same as in the group of women with anovulatory cycles. This could have detrimentally affected the power of the study in these subanalyses; hence, the results obtained from these groups should be interpreted with caution, and while taking into consideration the smaller sample size, they may still be used to inform and instigate further studies including subjects with similar characteristics. Furthermore, we did not perform blood tests to establish the concentrations of hormones across the menstrual cycle in order to determine the exact time of ovulation. Instead, we used a high-sensitivity self-administered urinary ovulation test. Additionally, since we repeated the same measurement procedure three or four times with the same participant, depending on the study group, the learning effect could have affected the results. Namely, in the group of women measured across the phases of one complete menstrual cycle, we detected improvements in most of the sensory performances (in nine out of 12 performed tests; 75%), starting from the mid-follicular phase towards the late luteal phase, which also coincided with the ordering of measurements. If this result was a mere consequence of the positive learning curve, it would have been present in other groups too, but that was not the case (in all of the five control groups only ten significant results across all tests were obtained; 16.7%). Since we have no reason to assume that the control groups would have a different learning curve than the 1mcW group, we can conclude that the changes observed in the 1mcW group were not due to the learning effect and were more likely attributable to the real effect of the menstrual cycle.

To the best of our knowledge, this is the first study to use such a broad methodological framework, helping us to provide answers to multiple hypotheses. We included as many as six distinct groups in the investigation of the effects of sex hormones, while exploring both olfactory and gustatory perception. Specifically, we could not find a single previously published study that had tested smell and taste senses within an anovulatory cycle in humans. Additionally, this study employed a dense measurement protocol within a longitudinal design and measurements were repeated with the same participants (increasing the power of the study). As many as four measurements were performed across distinct phases of the menstrual cycle. This has rarely been seen in the literature, as the majority of the studies have performed testing only in follicular and luteal phases. We included the mid-follicular phase due to the low concentrations of both estrogen and progesterone, the ovulation phase as a hormone-wise distinct and ascertainable phase (we used a self-administered urinary test), and the mid-luteal phase, which stands out as the progesterone-dominant phase. Additionally, we included the late luteal phase, which was infrequently represented in previous studies of sensory perception across the menstrual cycle. We were interested to see how this phase, characterized by a decrease in progesterone (and estrogen), is associated with olfactory and gustatory perception, given that decreased progesterone is regarded as a possible driver for premenstrual syndrome [80]. This is interesting due to the increased food cravings and emotional responses to foods during the late luteal phase recorded in women with premenstrual dysphoric disorder [81].

## 5. Conclusions

Our study confirms previous findings of varying sensory perception during different phases of a regular ovulatory menstrual cycle; however, we added some new discoveries to the body of knowledge. Our results demonstrated amelioration in both olfactory and gustatory perception over the course of the menstrual cycle, although only in women who were followed from the start of a menstrual cycle. The highest olfactory performance was observed at the end of the cycle. Taste hedonic and intensity ratings displayed a cyclic or linear pattern, most commonly peaking in perceived intensity and propensity in the mid-luteal phase. None of the other groups, even women who were tested four times across the same phases but belonging to two consecutive menstrual cycles, displayed such a pattern within their group. These findings point toward a specific effect of each menstrual cycle and its fine-tuned hormonal milieu. This should be taken into account in future studies of sensory acuity and perception, as well as in studies assessing food preferences and intake. Future studies should not only examine each individual cycle phase effect as an independent data point, but instead the complete menstrual cycle should be observed as a unit, starting with the follicular phase.

This study adds new insights into the complex mosaic behind the effects of the hypothalamic–pituitary–gonadal axis on the sensory physiology. Further studies are needed in order to confirm our results.

## Figures and Tables

**Table 1:** Characteristics of the participants.

	Men (M) *N* = 17	Postmenopausal Women (pmW) *N* = 14	Women Taking Oral Contraceptives (ocW) *N* = 10	Women with Anovulatory Cycle (aoW) *N* = 8	Women Across 1 Menstrual Cycle (1mcW) *N* = 21	Women Across 2 Menstrual Cycles (2mcW) *N* = 29	Overall *p* Value (between-Group Comparison)	Post hoc *p* Value (between-Group Comparison)
Age; median (IQR)	24.02 (6.24)	56.55 (3.45)	24.51 (1.50)	20.56 (16.26)	27.27 (7.87)	27.56 (12.49)	<0.001	<0.001 ^1,2^, <0.001 ^2,3^, <0.001 ^2,4^, <0.001 ^2,5^, <0.001 ^2,6^
BMI; median (IQR)	24.34 (2.38)	26.96 (3.93)	20.71 (3.79)	21.90 (5.68)	20.58 (2.09)	21.41 (2.22)	<0.001	0.017 ^1,2^, 0.009 ^1,3^, <0.001 ^1,5^, 0.001 ^1,6^, 0.001 ^2,3^, 0.017 ^2,4^, <0.001 ^2,5^, <0.001 ^2,6^
WHR; median (IQR)	0.84 (0.05)	0.86 (0.14)	0.71 (0.05)	0.76 (0.05)	0.73 (0.03)	0.73 (0.06)	<0.001	<0.001 ^1,3^, <0.001 ^1,4^, <0.001 ^1,5^, <0.001 ^1,6^, <0.001 ^2,3^, 0.006 ^2,4^, <0.001 ^2,5^, <0.001 ^2,6^
WtHR; median (IQR)	0.46 (0.05)	0.54 (0.09)	0.41 (0.04)	0.43 (0.08)	0.42 (0.02)	0.41 (0.07)	<0.001	<0.001 ^1,2^, 0.006 ^1,3^, <0.001 ^1,5^, 0.013 ^1,6^, <0.001 ^2,3^, 0.001 ^2,4^, <0.001 ^2,5^, <0.001 ^2,6^
SES; median (IQR)	3.50 (1.00)	4.00 (1.00)	3.00 (1.00)	3.00 (1.80)	3.50 (1.00)	4.00 (1.00)	0.330	Na
Smoking; n (%)							0.064	Na
Current	3 (17.60)	3 (21.40)	1 (10.00)	3 (37.50)	12 (57.10)	10 (34.50)		
Never/ex-smokers	14 (82.40)	11 (78.60)	9 (90.00)	5 (62.50)	9 (42.90)	19 (65.50)		

IQR - interquartile range; WHR—waist-to-hip ratio; WtHR—waist-to-height ratio; SES—socioeconomic status. Overall *p* values for between-group comparisons was obtained with Kruskal–Wallis test for numerical and Fisher’s exact test for categorical variables. Post hoc *p* values for between–group comparisons were obtained with the Mann–Whitney test (only significant *p* values are presented). ^1^ Men (M); ^2^ postmenopausal women (pmW); ^3^ oral contraceptive users (ocW); ^4^ women with an anovulatory cycle (aoW); ^5^ women measured in four consecutive phases of one complete menstrual cycle, starting with the mid-follicular phase (1mcW); ^6^ women measured in four phases belonging to two different, consecutive menstrual cycles (2mcW); Na—not applicable.

**Table 2:** Olfactory threshold, discrimination, identification test, and overall TDI scores (threshold, discrimination, and identification) for six groups of participants throughout measurement sessions.

	Men (M) *N* = 17	Postmenopausal Women (pmW) *N* = 14	Women Taking Oral Contraceptives (ocW) *N* = 10	Women with Anovulatory Cycle (aoW) *N* = 8	Women Across 1 Menstrual Cycle (1mcW) *N* = 21	Women Across 2 Menstrual Cycles (2mcW) * N = 29	Overall *p* Value (between-Groups Comparison)	Post hoc *p* Value (between-Group Comparison)
Threshold; mean ± SD								
1st measurement/mid-follicular	9.38 ± 2.13	8.95 ± 1.83	10.35 ± 1.63	11.50 ± 2.82	10.00 ± 1.69	10.28 ± 2.68	0.124	Na
2nd measurement/ovulation	10.71 ± 2.40	8.64 ± 2.81	10.60 ± 2.26	10.81 ± 1.96	10.58 ± 1.90	9.90 ± 2.94	0.183	Na
3rd measurement/mid-luteal	9.93 ± 1.57	10.64 ± 1.81	9.65 ± 2.67	10.19 ± 1.72	11.26 ± 1.72	9.84 ± 2.69	0.205	Na
4th measurement/late luteal					10.56 ± 1.80	9.60 ± 2.53	0.145	Na
Overall *p* value (within-group comparison)	0.098	F(2,26) = 5.84, partial ɳ^2^ = 0.31; 0.008	0.555	0.504	0.120	0.611		
Post hoc *p* value	Na	0.030^1st,3rd^, 0.012^2nd,3rd^	Na	Na	Na	Na		
Linear fit *p* value	0.731	0.423	0.497	0.017	0.410	0.038		
Trigonometric fit *p* value	-	-	-	-	0.366	0.432		
Discrimination; mean ± SD								
1st measurement/mid-follicular	12.24 ± 1.56	11.50 ± 1.65	12.60 ± 1.08	12.13 ± 1.89	12.05 ± 2.25	12.62 ± 1.92	0.530	Na
2nd measurement/ovulation	13.12 ± 1.27	10.50 ± 1.74	13.20 ± 1.69	13.25 ± 1.28	12.62 ± 1.56	13.14 ± 1.30	F(5,93) = 7.60, partial ɳ^2^ = 0.29; <0.001	<0.001 ^1,2^, <0.001 ^2,3^, 0.001 ^2,4^, 0.001 ^2,5^, <0.001 ^2,6^
3rd measurement/mid-luteal	12.82 ± 1.81	11.00 ± 1.78	13.60 ± 1.65	13.50 ± 1.60	13.14 ± 1.01	12.62 ± 1.78	F(5,93) = 4.39, partial ɳ^2^ = 0.19; 0.001	0.029 ^1,2^, 0.003 ^2,3^, 0.010 ^2,4^, 0.003 ^2,5^, 0.033 ^2,6^
4th measurement/late luteal					13.52 ± 1.25	12.76 ± 1.77	0.096	Na
Overall *p* value (within-group comparison)	0.235	0.233	0.314	0.124	F(3,60) = 3.87, partial ɳ^2^ = 0.16; 0.013	0.500		
Post hoc *p* value	Na	Na	Na	Na	-	Na		
Linear fit *p*-value	0.551	0.667	0.073	0.224	0.004	0.946		
Trigonometric fit *p*-value	-	-	-	-	0.138	0.950		
Identification; mean ± SD								
1st measurement/mid-follicular	13.24 ± 1.25	11.57 ± 1.83	13.70 ± 1.83	13.88 ± 1.136	12.67 ± 1.98	13.34 ± 1.72	F(5,93) = 3.20, partial ɳ^2^ = 0.15; 0.010	0.082 ^1,2^, 0.037 ^2,3^
2nd measurement/ovulation	13.47 ± 1.13	11.43 ± 1.51	14.00 ± 1.63	14.50 ± 1.20	13.38 ± 1.66	13.83 ± 1.28	F(5,93) = 7.35, partial ɳ^2^ = 0.28; <0.001	0.002 ^1,2^, <0.001 ^2,3^, <0.001 ^2,4^, 0.002 ^2,5^, <0.001 ^2,6^
3rd measurement/mid-luteal	13.53 ± 1.33	11.21 ± 2.08	14.90 ± 1.20	13.89 ± 1.55	13.52 ± 2.02	13.34 ± 1.40	F(5,93) = 6.77, partial ɳ^2^ = 0.27; <0.001	0.002 ^1,2^, <0.001 ^2,3^, 0.005 ^2,4^, 0.001 ^2,5^, 0.002 ^2,6^
4th measurement/late luteal					14.19 ± 1.50	13.48 ± 1.70	0.135	Na
Overall *p* value (within-group comparison)	0.647	0.766	0.036	0.360	F(3,60) = 8.20, partial ɳ^2^ = 0.29; <0.001	0.342		
Post hoc *p* value	Na	Na	-	Na	0.001^1st,4th^, 0.028^2nd,4th^	Na		
Linear fit *p* value	0.208	0.081	0.179	0.991	0.026	0.966		
Trigonometric fit *p* value	-	-	-	-	0.435	0.957		
TDI score; mean ± SD								
1st measurement/mid-follicular	34.85 ± 3.36	32.02 ± 3.33	36.65 ± 2.30	37.50 ± 4.65	34.71 ± 3.99	36.09 ± 3.84	F(5,93) = 3.48, partial ɳ^2^ = 0.16; 0.006	0.035 ^2,3^, 0.014 ^2,4^, 0.012 ^2,6^
2nd measurement/ovulation	37.29 ±3.21	30.57 ± 4.20	37.80 ± 3.84	38.56 ± 2.68	36.58 ± 3.79	36.86 ± 3.48	F(5,93) = 8.47, partial ɳ^2^ = 0.31; <0.001	<0.001 ^1,2^, <0.001 ^2,3^, <0.001 ^2,4^, <0.001 ^2,5^, <0.001 ^2,6^
3rd measurement/mid-luteal	36.28 ± 3.47	32.86 ± 4.47	38.15 ± 3.16	37.56 ± 3.01	37.93 ± 2.92	35.80 ± 3.06	F(5,93) = 4944, partial ɳ^2^ = 0.21; <0.001	0.003 ^2,3^, 0.024 ^2,4^, <0.001 ^2,5^, 0.003 ^2,6^
4th measurement/late luteal					38.27 ± 2.36	35.85 ± 3.57	F(5,93) = 7.37, partial ɳ^2^ = 0.13; 0.009	Na
Overall *p* value (within-group comparison)	F(2,32) = 3.93, partial ɳ^2^ = 0.20; 0.030	0.085	0.430	0.797	F(3,60) = 7.72, partial ɳ^2^ = 0.28; <0.001	0.425		
Post hoc *p* value	0.028^1st,2nd^	Na	Na	Na	0.004^1st,3rd^, 0.005^1st,4th^	Na		
Linear fit *p* value	0.605	0.764	0.192	0.966	0.039	0.532		
Trigonometric fit *p* value	-	-	-	-	0.106	0.818		

* For 2mcW group, the order of data does not follow the actual timeline of measurement sessions (unlike all other groups), but instead the phases of the menstrual cycle. The 1st measurement denotes the mid-follicular phase, 2nd is ovulation, 3rd is mid-luteal, and 4th measurement is the late luteal phase. Overall *p* values for between-group comparisons were obtained with one-way ANOVA. Post hoc *p* values for between-group comparison were obtained with Tukey’s HSD post hoc test. ^1^ Men (M); ^2^ postmenopausal women (pmW); ^3^ oral contraceptive users (ocW); ^4^ women with an anovulatory cycle (aoW); ^5^ women measured in four consecutive phases of one complete menstrual cycle, starting with the mid-follicular phase (1mcW); ^6^ women measured in four phases belonging to two different and consecutive menstrual cycles (2mcW). Overall *p* value for within-group comparisons was obtained with ANOVA for repeated measures (sphericity assumed). Post hoc *p* values for within-group pairwise comparison were based on estimated marginal means, adjustment for multiple comparisons—Bonferroni for i^th^ and j^th^ sampling time: 1st measurement or mid-follicular phase; 2nd measurement or ovulation; 3rd measurement or mid-luteal phase; 4th measurement or late luteal phase; Na—not applicable.

**Table 3:** Differences in olfactory perception across study groups and measurements or menstrual cycle phases, using linear mixed models controlled for age, smoking, and waist-to-height ratio.

	Olfactory Threshold Estimate (95% CI); P	Olfactory Discrimination Estimate (95% CI); P	Olfactory Identification Estimate (95% CI); P	TDI Score Estimate (95% CI); P
Group (women across 2 cycles are referent group)				
Men (M)	−0.05 (−1.43–1.34); 0.945	0.07 (−0.91–1.05); 0.893	0.49 (−0.44–1.42); 0.297	0.52 (−1.61–2.64); 0.632
Postmenopausal women (pmW)	1.33 (−1.06–3.74); 0.273	−1.02 (−2.59–0.55); 0.203	−2.53 (−4.22–−0.85); 0.004	−2.21 (−5.94–1.51); 0.242
Women taking oral contraceptives (ocW)	−0.07 (−1.75–1.63); 0.939	0.74 (−0.46–1.94); 0.225	1.79 (0.65–2.93); 0.002	2.47 (−1.13–5.06); 0.062
Women with anovulatory cycle (aoW)	0.43 (−1.40–2.26); 0.643	0.71 (0.59–2.01); 0.281	0.58 (−0.65–1.81); 0.352	1.73 (−1.08–4.54); 0.226
Women across 1 menstrual cycle (1mcW)	1.10 (−0.25–2.45); 0.109	0.84 (−1.12–1.79); 0.087	0.88 (−0.18–1.79); 0.055	2.81 (0.74–4.87); 0.008
Time (last measurement is referent group)				
1st measurement/mid-follicular	0.90 (−0.04–1.84); 0.059	−0.30 (−1.07–0.47); 0.448	−0.14 (−0.70–0.41); 0.608	0.32 (−1.08–1.71); 0.655
2nd measurement/ovulation	0.22 (−0.72–1.16); 0.641	0.36 (−0.41–1.13); 0.363	0.37 (−0.18–0.93); 0.189	0.96 (−0.43–2.36); 0.175
3rd measurement/mid-luteal	0.34 (−0.60–1.28); 0.472	−0.06 (−0.83–0.71); 0.886	−0.07 (−0.63–0.48); 0.788	0.22 (−1.17–1.62); 0.754
Group*time (the last measurement is referent group)				
Men*1st measurement	−0.85 (−2.36–0.65); 0.265	−0.31 (−1.55–0.92); 0.617	−0.38 (−1.26–0.51); 0.406	−1.39 (−3.62–0.84); 0.222
Men*2nd measurement	1.26 (−0.24–2.76); 0.100	−0.08 (−1.32–1.15); 0.898	−0.61 (−1.50–0.27); 0.174	0.56 (−1.67–2.80); 0.619
Postmenopausal women (pmW)*1st measurement	−2.21 (−3.88–−0.54); 0.010	0.86 (−0.52–2.23); 0.221	0.84 (−0.15–1.83); 0.096	−0.36 (−2.85–2.12); 0.773
Postmenopausal women (pmW)*2nd measurement	−1.96 (−3.63–−0.28); 0.022	−0.64 (−2.02–0.73); 0.356	0.01 (−0.98–1.00); 0.979	−2.59 (−5.07–−0.11); 0.041
Women taking oral contraceptives (ocW)*1st measurement	0.14 (−1.70–1.98); 0.881	−0.76 (−2.27–0.75); 0.323	−1.13 (−2.22–−0.04); 0.041	−1.59 (−4.32–1.31); 0.250
Women taking oral contraceptives (ocW)*2nd measurement	1.07 (−0.76–2.91); 0.252	−0.81 (−2.32–0.70); 0.289	−1.35 (−2.43–−0.26); 0.015	−1.09 (−3.82–1.64); 0.431
Women with anovulatory cycle (aoW)*1st measurement	0.75 (−1.25–2.75); 0.459	−1.13 (−2.78–0.51); 0.176	0.07 (−1.11–1.25); 0.909	−0.15 (−3.13–2.81); 0.917
Women with anovulatory cycle (aoW)*2nd measurement	0.75 (−1.25–2.74); 0.463	−0.66 (−2.31–0.98); 0.427	0.18 (−1.00–1.36); 0.769	0.26 (−2.71–3.23); 0.864
Women across 1 menstrual cycle (1mcW)*1st measurement	−1.47 (−2.91–−0.2); 0.047	−1.26 (−2.45–−0.07); 0.038	−1.37 (−2.22–−0.51); 0.002	−3.93 (−6.08–−1.78); <0.001
Women across 1 menstrual cycle (1mcW)*2nd measurement	−0.52 (−1.97–0.92); 0.477	−1.37 (−2.56–−0.18); 0.024	−1.16 (−2.01–−0.30); 0.008	−3.04 (−5.19–−0.89); 0.006
Women across 1 menstrual cycle (1mcW)*3rd measurement	0.38 (−1.07–1.83); 0.606	−0.41 (−1.60–0.78); 0.496	−0.62 (−1.47–0.24); 0.157	−0.64 (−2.79–1.51); 0.556
Age	−0.02 (−0.08–0.04); 0.462	−0.04 (−0.07–−0.01); 0.046	0.01 (−0.03–0.05); 0.677	−0.05 (−0.14–0.04); 0.292
Smoking (yes is referent group)	−0.14 (−0.96–0.67); 0.730	0.25 (−0.25–0.75); 0.319	−0.57 (−1.17–0.17); 0.057	−0.48 (−1.76–0.80); 0.455
WtHR (elevated is referent group)	−0.18 (−1.23–0.87); 0.733	−0.25 (−0.90–0.40); 0.443	−0.09 (−0.75–0.67); 0.819	−0.50 (−2.15–1.15); 0.547

WtHR—waist-to-height ratio.

**Table 4:** Taste intensity and hedonic ratings for sweet (sucrose), salty (NaCl), sour (ascorbic acid), and bitter (quinine HCl) solutions in six groups of participants throughout measurement sessions.

	Men (M) *N* = 16	Postmenopausal Women (pmW) *N* = 14	Women Taking Oral Contraceptives (ocW) *N* = 8	Women with Anovulatory Cycle (aoW) *N* = 8	Women Across 1 Menstrual Cycle (1mcW) *N* = 18	Women Across 2 Menstrual Cycles (2mcW) * N = 29	Overall *p* Value (between-Group Comparison)	Post hoc *p* Value (between-Group Comparison)
Sweet taste intensity (mm); mean ± SD								
1st measurement/mid-follicular	63.69 ± 26.79	38.50 ± 26.47	26.38 ± 26.75	44.25 ± 21.04	48.89 ± 17.58	55.14 ± 21.21	F(5,87) = 4.34, partial ɳ^2^ = 0.20; 0.001	0.026 ^1,2^, 0.002 ^1,3^, 0.017 ^3,6^
2nd measurement/ovulation	62.56 ± 25.83	48.79 ± 30.29	34.38 ± 9.94	59.50 ± 32.81	53.56 ± 25.63	52.03 ± 23.32	0.200	Na
3rd measurement/mid-luteal	60.31 ± 23.60	43.00 ± 15.47	45.88 ± 21.30	43.25 ± 28.57	61.83 ± 26.52	47.86 ± 20.02	0.068	Na
4th measurement/late luteal					53.61 ± 26.25	54.62 ± 20.46	0.883	Na
Overall *p* value (within-group comparison)	0.746	0.282	F(2,14) = 8.87, partial ɳ^2^ = 0.56; 0.003	0.148	0.142	0.362		
Post hoc *p* value	Na	Na	0.018^1st,3rd^	Na	Na	Na		
Linear fit *p* value	0.121	0.713	0.066	0.965	0.462	0.779		
Trigonometric fit *p* value	-	-	-	-	0.507	0.535		
Sweet taste hedonics (mm); mean ± SD								
1st measurement/mid-follicular	20.31 ± 19.93	−3.29 ± 22.29	12.00 ± 19.50	8.88 ± 18.07	3.56 ± 20.84	9.69 ± 20.15	0.051	Na
2nd measurement/ovulation	24.13 ± 14.46	3.71 ± 18.61	15.00 ± 19.40	13.88 ± 16.94	8.50 ± 19.41	8.79 ± 20.36	0.051	Na
3rd measurement/mid-luteal	24.25 ± 13.13	0.86 ± 19.37	13.50 ± 16.51	18.00 ± 10.93	12.67 ± 15.35	8.04 ± 18.78	F(5,87) = 3.55, partial ɳ^2^ = 0.17; 0.006	0.003 ^1,2^, 0.028 ^1,6^
4th measurement/late luteal					12.44 ± 10.97	10.41 ± 18.59	0.677	Na
Overall *p* value (within-group comparison)	0.388	0.610	0.517	0.328	0.150	0.938		
Linear fit *p* value	0.316	0.599	0.667	0.035	0.069	0.824		
Trigonometric fit *p* value	-	-	-	-	0.011	0.486		
Salt taste intensity (mm); mean ± SD								
1st measurement/mid-follicular	83.81 ± 26.94	56.21 ± 22.33	59.00 ± 11.86	59.75 ± 31.83	63.83 ± 23.70	71.38 ± 26.90	F(5,87) = 2.45, partial ɳ^2^ = 0.12; 0.040	0.041 ^1,2^
2nd measurement/ovulation	83.06 ± 25.64	54.00 ± 24.45	64.75 ± 14.45	71.38 ± 36.88	77.94 ± 26.52	75.38 ± 23.57	F(5,87) = 2.43, partial ɳ^2^ = 0.12; 0.041	0.028 ^1,2^
3rd measurement/mid-luteal	73.00 ± 27.53	59.07 ± 26.57	73.00 ± 19.82	66.13 ± 27.46	82.33 ± 22.72	71.10 ± 28.01	0.256	Na
4th measurement/late luteal					71.78 ± 25.18	78.48 ± 20.84	0.328	Na
Overall *p* value (within-group comparison)	0.175	0.792	0.115	0.545	F(3,51) = 5.20, partial ɳ^2^ = 0.23; 0.003	0.477		
Post hoc *p* value	Na	Na	Na	Na	0.019^1st,3rd^	Na		
Linear fit *p* value	0.297	0.620	0.065	0.631	0.546	0.376		
Trigonometric fit *p* value	-	-	-	-	0.033	0.899		
Salt taste hedonics (mm); mean ± SD								
1st measurement/mid-follicular	−20.06 ± 21.64	−11.88± 17.94	−22.13 ± 16.69	−9.63 ± 24.84	−22.56 ± 21.51	−14.66 ± 24.16	0.564	Na
2nd measurement/ovulation	−11.63 ± 22.23	−16.14 ± 22.99	−12.63 ± 23.76	−8.38 ± 21.30	−7.44 ± 26.75	−11.45 ± 23.06	0.942	Na
3rd measurement/mid-luteal	−1.00 ± 25.83	−22.07 ± 17.66	−18.75 ± 16.70	0.25 ± 18.14	−6.33 ± 26.37	−11.69 ± 22.40	0.085	Na
4th measurement/late luteal					−3.17 ± 25.07	−12.45 ± 24.95	0.222	Na
Overall *p* value (within-group comparison)	F(1.35,20.33) = 5.56, partial ɳ^2^ = 0.27; 0.020	0.350	0.357	0.303	F(1.86,31.55) = 9.02, partial ɳ^2^ = 0.35; 0.001	0.727		
Post hoc *p* value	0.042^2nd,3rd^	Na	Na	Na	0.023^1st,3rd^, 0.007^1st,4th^	Na		
Linear fit *p* value	0.042	0.060	0.772	0.259	0.115	0.436		
Trigonometric fit *p* value	-	-	-	-	0.435	0.410		
Sour taste intensity (mm); mean ± SD								
1st measurement/mid-follicular	65.56 ± 32.408	50.00 ± 23.57	56.38 ± 24.75	63.50 ± 34.42	62.11 ± 26.43	66.45 ± 25.95	0.550	Na
2nd measurement/ovulation	75.81 ± 24.40	54.64 ± 22.38	66.00 ± 29.34	66.88 ± 29.56	77.11 ± 29.50	71.55 ± 24.47	0.205	Na
3rd measurement/mid-luteal	69.19 ± 26.61	54.50 ± 23.56	77.25 ± 26.91	71.38 ± 25.81	74.39 ± 24.57	61.41 ± 21.91	0.135	Na
4th measurement/late luteal					69.17 ± 31.86	71.69 ± 28.35	0.779	Na
Overall *p* value (within-group comparison)	0.293	0.584	0.119	0.734	F(3,51) = 3.00, partial ɳ^2^ = 0.15; 0.039	0.148		
Post hoc *p* value	Na	Na	Na	Na	0.048^1st,3rd^	Na		
Linear fit *p* value	0.773	0.305	0.029	0.052	0.639	0.853		
Trigonometric fit *p* value	-	-	-	-	0.425	0.987		
Sour taste hedonics (mm); mean ± SD								
1st measurement/mid-follicular	4.94 ± 21.33	−9.21± 14.30	−19.88 ± 19.59	0.63 ± 19.326	−3.06 ± 29.97	−5.93 ± 17.69	0.129	Na
2nd measurement/ovulation	4.00 ± 23.06	−9.64 ± 21.58	−9.88 ± 28.63	−3.63 ± 20.02	−0.17 ± 25.40	−2.45 ± 20.45	0.594	Na
3rd measurement/mid-luteal	5.00 ± 22.21	−1.79 ± 22.04	−23.00 ± 18.72	3.87 ± 13.10	2.00 ± 28.20	−6.04 ± 20.40	0.064	Na
4th measurement/late luteal					5.83 ± 24.16	−0.10 ± 21.99	0.391	Na
Overall *p* value (within-group comparison)	0.954	0.436	0.405	0.331	0.243	0.169		
Linear fit *p* value	0.965	0.364	0.854	0.716	0.006	0.377		
Trigonometric fit *p* value	-	-	-	-	0.261	0.908		
Bitter taste intensity (mm); mean ± SD								
1st measurement/mid-follicular	74.25 ± 29.93	59.71 ± 35.76	45.00 ± 22.56	56.88 ± 27.73	67.50 ± 35.94	73.45 ± 33.68	0.232	Na
2nd measurement/ovulation	84.06 ± 25.88	60.64 ± 35.22	58.38 ± 21.69	61.13 ± 33.05	70.94 ± 37.17	74.93 ± 33.96	0.288	Na
3rd measurement/mid-luteal	80.69 ± 25.60	58.29 ± 36.51	65.88 ± 27.49	72.63 ± 33.98	72.06 ± 34.75	65.83 ± 36.05	0.558	Na
4th measurement/late luteal					75.22 ± 28.23	72.38 ± 31.66	0.757	Na
Overall *p* value within-group comparison)	0.254	0.968	0.247	0.270	0.669	0.414		
Linear fit *p* value	0.553	0.589	0.103	0.165	0.016	0.604		
Trigonometric fit *p* value	-	-	-	-	0.375	0.886		
Bitter taste hedonics (mm); mean ± SD								
1st measurement/mid-follicular	−33.75 ± 11.13	−21.54 ± 19.29	−31.38 ± 10.85	−27.88 ± 18.79	−28.00 ± 18.48	−33.79 ± 12.17	0.185	Na
2nd measurement/ovulation	−28.81 ± 16.71	−28.36 ± 18.38	−30.00 ± 16.18	−25.50 ± 14.51	−26.28 ± 24.53	−33.79 ± 12.99	0.724	Na
3rd measurement/mid-luteal	−28.44 ± 16.90	−24.43 ± 18.58	−34.50 ± 11.75	−34.13 ± 12.29	−26.56 ± 23.07	−24.28 ± 20.07	0.648	Na
4th measurement/late luteal					−25.89 ± 21.88	−30.17 ± 14.92	0.429	Na
Overall *p* value (within-group comparison)	0.107	0.571	0.543	0.255	0.893	F(1.94,54.37) = 3.9, partial ɳ^2^ = 0.13; 0.025		
Post hoc *p* value	Na	Na	Na	Na	Na	-		
Linear fit *p* value	0.293	0.722	0.526	0.505	0.151	0.414		
Trigonometric fit *p* value	-	-	-	-	0.627	0.752		

Note: * For 2mcW group, the ordering of the data does not follow the actual timeline of measurement sessions (as it does for all other groups), but instead the phases of menstrual cycle. The 1st measurement denotes the mid-follicular phase, 2nd is ovulation, 3rd is mid-luteal, and 4th the is late luteal phase. Overall *p* value for between-group comparisons was obtained with one-way ANOVA. Post hoc *p* values for between-group comparison were obtained with Tukey’s HSD post hoc test. ^1^ Men (M); ^2^ postmenopausal women (pmW); ^3^ oral contraceptive users (ocW); ^4^ women with an anovulatory cycle (aoW); ^5^ women measured in four consecutive phases of one complete menstrual cycle, starting with mid-follicular phase (1mcW); ^6^ women measured in four phases belonging to two different, consecutive menstrual cycles (2mcW). Overall *p* value for within-group comparisons was obtained with ANOVA for repeated measures (sphericity assumed or Greenhouse–Geisser correction). Post hoc *p* values for within-group pairwise comparison were based on estimated marginal means, with adjustment for multiple comparisons, including Bonferroni correction for ith and jth sampling times; 1st measurement or mid-follicular phase; 2nd measurement or ovulation; 3rd measurement or mid-luteal phase; 4th measurement or late luteal phase; Na—not applicable.

**Table 5:** Differences in gustatory intensity perception across study groups and measurements or menstrual cycle phases, using linear mixed models controlled for age, smoking, and waist-to-height ratio.

	Sweet taste Intensity Estimate (95% CI); P	Salt Taste Intensity Estimate (95% CI); P	Sour Taste Intensity Estimate (95% CI); P	Bitter Taste Intensity Estimate (95% CI); P
Group (Women across 2 menstrual cycles are referent group)				
Men (M)	6.90 (−4.33–24.13); 0.171	−0.85 (−16.60–14.90); 0.915	5.01 (−11.78–21.79); 0.557	12.63 (−7.05–32.31); 0.207
Postmenopausal women (pmW)	−17.21 (−42.43–8.00); 0.179	−14.03 (−41.70–13.65); 0.317	−2.10 (−32.47–28.27); 0.891	1.57 (−34.11–37.25); 0.931
Women taking oral contraceptives (ocW)	−1.47 (−19.66–16.72); 0.873	0.29 (−19.85–20.42); 0.978	12.84 (−8.62–34.30); 0.239	−4.83 (−29.99–20.33); 0.705
Women with anovulatory cycle (aoW)	−5.48 (−23.55–15.59); 0.550	−7.16 (−27.17–12.84); 0.481	8.54 (−12.77–29.84); 0.430	6.66 (−18.32–31.63); 0.599
Women across 1 menstrual cycle (1mcW)	1.34 (−12.69–15.38); 0.850	−5.29 (−20.82–10.25); 0.503	0.95 (−15.62–17.51); 0.910	9.61 (−9.81–29.03); 0.330
Time (last measurement is referent group)				
1st measurement/mid-follicular	0.53 (−8.05–9.11); 0.902	−7.32 (−17.06–2.42); 0.140	−5.43 (−14.90–4.05); 0.260	1.18 (−9.84–12.20); 0.833
2nd measurement/ovulation	−3.43 (−12.01–5.15); 0.432	−2.54 (−12.27–7.20); 0.608	0.46 (−9.01–9.94); 0.923	2.61 (−8.42–13.63); 0.642
3rd measurement/mid-luteal	−7.75 (−16.33–0.83); 0.076	−6.89 (−16.63–2.84); 0.164	−9.89 (−19.37–−0.42); 0.041	−4.18 (−15.20–6.84); 0.456
Group*time (the last measurement is referent group)				
Men*1st measurement	−4.91 (−19.14–9.32); 0.497	11.24 (−4.91–27.39); 0.171	−8.09 (−23.80–7.62); 0.311	−11.79 (−30.07–6.49); 0.205
Men*2nd measurement	−2.07 (−16.30–12.15); 0.774	5.71 (−10.44–21.85); 0.487	−3.73 (−19.44–11.98); 0.640	−3.41 (−21.69–14.87); 0.713
Postmenopausal women (pmW)*1st measurement	−11.67 (−26.91–3.57); 0.133	−1.42 (−18.71–15.88); 0.872	−9.39 (−26.21–7.44); 0.273	−4.66 (−24.24–14.91); 0.639
Postmenopausal women (pmW)*2nd measurement	−2.09 (−17.33–13.15); 0.787	−12.43 (−29.73–4.86); 0.158	−10.28 (−27.11–6.55); 0.230	−5.09 (−24.67–14.48); 0.609
Women taking oral contraceptives (ocW)*1st measurement	−27.78 (−45.99–−9.58); 0.003	−13.57 (−34.23–7.08); 0.197	−25.34 (−45.44–−5.24); 0.014	−26.23 (−49.62–−2.85); 0.028
Women taking oral contraceptives (ocW)*2nd measurement	−15.82 (−34.03–2.38); 0.088	−12.61 (−33.26–8.05); 0.230	−21.61 (−41.71–−1.51); 0.035	−14.29 (−37.67–9.10); 0.230
Women with anovulatory cycle (aoW)*1st measurement	−7.29 (−25.49–10.92); 0.431	−5.95 (−26.60–14.71); 0.571	−12.34 (−32.45–7.76); 0.228	−21.11 (−44.49–2.28); 0.077
Women with anovulatory cycle (aoW)*2nd measurement	11.93 (−6.28–30.13); 0.198	0.89 (−19.76–21.55); 0.932	−14.86 (−34.9–5.24); 0.147	−18.29 (−41.67–5.10); 0.125
Women across 1 menstrual cycle (1mcW)*1st measurement	−3.12 (−17.09–10.84); 0.660	−1.09 (−16.93–14.75); 0.892	−3.81 (−19.22–11.61); 0.627	−9.36 (−27.29–8.58); 0.305
Women across 1 menstrual cycle (1mcW)*2nd measurement	4.55 (−9.42–18.51); 0.522	9.07 (−6.78–24.91); 0.261	4.18 (−11.23–19.60); 0.593	−7.20 (−25.13–10.74); 0.430
Women across 1 menstrual cycle (1mcW)*3rd measurement	17.69 (3.73–31.65); 0.013	15.72 (−0.13–31.56); 0.052	14.19 (−1.23–29.60); 0.071	1.88 (−16.05–19.82); 0.836
Age	0.01 (−0.63–0.65); 0.982	−0.23 (−0.93–0.47); 0.518	−0.28 (−1.06–0.50); 0.472	−0.10 (−1.02–0.81); 0.823
Smoking (yes is referent group)	8.91 (0.10–17.73); 0.047	4.31 (−5.31–13.92); 0.376	8.78 (−1.96–19.52); 0.108	11.73 (−0.90–24.36); 0.068
WtHR (elevated is referent group)	−14.40 (−25.82–−2.99); 0.014	−7.70 (−20.17–4.77); 0.223	−3.84 (−17.74–10.06); 0.584	6.72 (−9.63–23.07); 0.416

WtHR—waist-to-height ratio.

**Table 6:** Differences in gustatory hedonic perception across study groups and measurements or menstrual cycle phases, using linear mixed models controlled for age, smoking, and waist-to-height ratio.

	Sweet Taste Hedonics Estimate (95% CI); P	Salt Taste Hedonics Estimate (95% CI); P	Sour Taste Hedonics Estimate (95% CI); P	Bitter Taste Hedonics Estimate (95% CI); P
Group (Women across 2 menstrual cycles are referent group)				
Men (M)	14.05 (2.90–25.19); 0.014	9.33 (−4.89–23.55); 0.197	11.12 (−2.60–24.84); 0.111	−6.54 (−16.94–3.86); 0.216
Postmenopausal women (pmW)	−5.10 (−24.33–14.12); 0.600	1.74 (−24.72–28.20); 0.896	4.84 (−20.64–30.32); 0.707	−8.26 (−27.01–10.48); 0.385
Women taking oral contraceptives (ocW)	3.97 (−10.27–18.22); 0.583	−10.17 (−28.35–8.02); 0.271	−15.67 (−33.21–1.87); 0.079	−10.27 (−23.68–3.13); 0.132
Women with anovulatory cycle (aoW)	7.59 (−6.57–21.75); 0.292	11.37 (−6.66–29.41); 0.215	9.82 (−7.58–27.22); 0.266	−11.07 (−24.74–2.59); 0.112
Women across 1 menstrual cycle (1mcW)	3.21 (−7.78–14.20); 0.565	11.05 (−2.99–25.09); 0.122	7.65 (−5.89–21.19); 0.266	1.16 (−8.95–11.27); 0.821
Time (last measurement is referent group)				
1st measurement/mid-follicular	0.54 (−6.68–7.75); 0.884	−1.29 (−8.46–5.88); 0.724	−5.21 (−12.18–1.75); 0.141	−3.05 (−9.02–2.91); 0.315
2nd measurement/ovulation	−0.07 (−7.29–7.15); 0.984	1.57 (−5.60–8.74); 0.666	−2.11 (−9.07–4.86); 0.551	−3.19 (−9.15–2.78); 0.294
3rd measurement/mid-luteal	−0.50 (−7.72–6.72); 0.891	1.43 (−5.74–8.60); 0.695	−6.14 (−13.11–0.82); 0.083	6.18 (0.21–12.15); 0.042
Group*time (the last measurement is referent group)				
Men*1st measurement	−4.97 (−16.94–6.99); 0.414	−16.35 (−28.24–−4.46); 0.007	−0.99 (−12.54–10.56); 0.866	4.12 (−5.64–13.88); 0.407
Men*2nd measurement	−0.55 (−12.52–11.41); 0.927	−10.77 (−22.66–1.12); 0.076	−5.04 (−16.58–6.51); 0.391	9.30 (−0.46–19.06); 0.062
Postmenopausal women (pmW)*1st measurement	−3.04 (−15.84–9.78); 0.641	16.10 (3.37–28.83); 0.013	−6.39 (−18.76–5.98); 0.310	15.73 (4.99–26.47); 0.004
Postmenopausal women (pmW)*2nd measurement	2.65 (−10.17–15.46); 0.684	6.93 (−5.80–19.67); 0.284	−12.57 (−24.94–−0.21); 0.046	6.83 (−3.91–17.57); 0.211
Women taking oral contraceptives (ocW)*1st measurement	−2.54 (−17.84–12.77); 0.744	−0.67 (−15.87–15.55); 0.932	2.20 (−12.57–16.97); 0.770	12.04 (−0.60–24.68); 0.062
Women taking oral contraceptives (ocW)*2nd measurement	1.07 (−14.24–16.38); 0.890	5.98 (−9.23–21.19); 0.439	9.09 (−5.68–23.86); 0.226	13.87 (10.00–26.73); 0.035
Women with anovulatory cycle (aoW)*1st measurement	−10.16 (−25.47–5.15); 0.192	−7.16 (−22.37–8.05); 0.354	−4.18 (−18.95–10.59); 0.578	15.48 (2.62–28.35); 0.019
Women with anovulatory cycle (aoW)*2nd measurement	−4.55 (−19.86–10.75); 0.558	−8.77 (−23.98–6.44); 0.257	−11.54 (−26.31–3.23); 0.125	17.99 (5.12–30.86); 0.006
Women across 1 menstrual cycle (1mcW)*1st measurement	−9.95 (−21.69–1.79); 0.096	−16.71 (−28.38–−5.05); 0.005	−1.61 (−12.94–9.72); 0.780	3.63 (−5.74–13.01); 0.446
Women across 1 menstrual cycle (1mcW)*2nd measurement	−5.11 (−16.85–6.64); 0.392	−3.87 (−15.53–7.80); 0.514	−1.78 (−13.10–9.55); 0.758	4.26 (−5.28–13.81); 0.380
Women across 1 menstrual cycle (1mcW)*3rd measurement	−0.26 (−12.01–11.48); 0.965	−2.90 (−14.56–8.77); 0.625	4.67 (−6.66–16.00); 0.417	−6.37 (−15.75–3.00); 0.182
Age	−0.18 (−0.66–0.30); 0.448	−0.43 (−1.12–0.26); 0.219	−0.16 (−0.83–0.50); 0.625	0.09 (−0.39–0.56); 0.721
Smoking (yes is referent group)	−0.05 (−6.66–6.55); 0.987	6.67 (−2.83–16.17); 0.166	0.01 (−9.14–9.15); 0.999	−0.86 (−7.48–5.76); 0.797
WtHR (elevated is referent group)	−4.37 (−12.94–4.20); 0.313	1.43 (−10.85–13.71); 0.817	−6.32 (−18.14–5.50); 0.291	−5.00 (−13.37–3.37); 0.239

WtHR–waist-to-height ratio.

## Data Availability

The datasets generated and analyzed during the current study are available from the corresponding author upon reasonable request.

## Supplementary Materials

The following are available online at https://www.mdpi.com/article/10.3390/nu13082509/s1: Table S1: Hunger, thirst, and emotional state ratings (stress, anxiety, happiness) in six groups of participants.
