# Supplementary material for: Does Each Menstrual Cycle Elicit a Distinct Effect on Olfactory and Gustatory Perception?
Source: Nutrients. 2021 Jul 22;13(8):2509. doi: 10.3390/nu13082509 (PMC8401541; doi:10.3390/nu13082509)
Supplement: Supplementary file 1 [file nutrients-13-02509-s001.zip › nutrients-1308356-supplementary.pdf]

nutrients

MDPI

Supplementary Table 1. Hunger and thirst rating, and emotional state rating (stress, anxiety, happiness) in six groups of participants

	Men (M) N=17	Postmenopausal women (pmW) N=14	Women taking oral contraceptives (ocW) N=10	Women with anovulatory cycle (aoW) N=8	Women across 1 menstrual cycle (1mcW) N=21	Women across 2 menstrual cycles (2mcW)* N=29	Overall P value (between groups comparison)	*Post-hoc* P value (between groups comparison)
**Hunger rating; median (IQR)**								
1st measurement/mid-follicular	3.50 (4.25)	5.00 (2.75)	5.00 (1.75)	4.00 (6.00)	7.00 (4.25)	4.00 (4.50)	0.120	Na
2nd measurement/ovulation	4.50 (3.75)	3.00 (4.50)	3.00 (4.00)	3.00 (3.00)	5.00 (5.00)	5.00 (4.00)	0.379	Na
3rd measurement/mid-luteal	4.50 (3.00)	5.00 (5.00)	5.00 (5.00)	6.00 (2.75)	6.00 (5.00)	5.00 (5.00)	0.425	Na
4th measurement/late luteal					7.00 (2.50)	4.00 (3.00)	0.059	Na
**Overall P value**	0.459	0.607	0.651	0.385	0.202	0.680		
**Thirst rating; median (IQR)**								
1st measurement/mid-follicular	4.00 (3.00)	3.50 (2.75)	5.00 (2.00)	4.00 (7.00)	4.00 (4.00)	6.00 (3.00)	0.063	Na
2nd measurement/ovulation	4.00 (3.00)	4.00 (7.75)	5.00 (4.00)	4.50 (4.75)	6.00 (4.00)	6.00 (4.00)	0.177	Na
3rd measurement/mid-luteal	6.00 (2.00)	4.50 (5.25)	7.00 (3.00)	5.00 (2.00)	6.00 (4.00)	5.00 (3.00)	0.128	Na
4th measurement/late luteal					6.00 (2.75)	5.50 (5.00)	0.716	Na
**Overall P value**	0.057	0.326	0.102	0.861	0.309	0.037		
**Post-hoc P value**	Na	Na	Na	Na	Na	0.029[13], 0.030[23]		
**Stress; median (IQR)**								
1st measurement/mid-follicular	2.00 (2.00)	3.00 (3.00)	2.00 (3.00)	2.00 (2.00)	2.00 (3.00)	2.00 (4.00)	0.324	Na
2nd measurement/ovulation	2.00 (2.00)	3.50 (2.00)	3.50 (6.00)	1.50 (2.00)	3.50 (4.00)	3.00 (3.00)	0.036	0.004[12], 0.002[24], 0.039[34]
3rd measurement/mid-luteal	2.00 (2.00)	4.00 (2.00)	3.00 (6.00)	2.50 (2.00)	2.00 (3.00)	3.00 (5.00)	0.072	Na
4th measurement/late luteal					3.00 (4.00)	3.00 (4.00)	0.754	Na
**Overall P value**	0.199	0.401	0.177	0.350	0.419	0.409		
**Anxiety; median (IQR)**								
1st measurement/mid-follicular	2.00 (2.00)	3.00 (1.00)	1.00 (3.00)	1.50 (4.00)	1.00 (2.00)	2.00 (2.00)	0.134	Na

2nd measurement/ovulation	2.00 (1.00)	4.00 (3.00)	4.00 (3.00)	2.00 (2.00)	1.00 (2.00)	2.00 (2.00)	0.028	0.003[12], 0.022[24], 0.002[25], 0.010[26]
3rd measurement/mid-luteal	2.00 (1.00)	4.00 (2.00)	3.00 (4.00)	1.50 (1.00)	1.00 (2.00)	2.00 (2.00)	<0.001	<0.001[12], 0.012[23], 0.001[24], <0.001[25], 0.003[26], 0.042[34], 0.019[35]
4th measurement/late luteal					1.00 (2.00)	2.00 (3.00)	0.676	Na
**Overall P value**	0.217	0.205	0.125	0.538	0.986	0.764		
**Happiness; median (IQR)**								
1st measurement/mid-follicular	7.00 (3.00)	7.00 (4.00)	8.00 (3.00)	8.00 (3.00)	9.00 (1.00)	8.00 (2.00)	0.309	Na
2nd measurement/ovulation	8.00 (2.00)	6.50 (3.00)	7.00 (3.00)	8.00 (2.00)	9.00 (1.00)	8.00 (2.00)	0.002	0.021[12], 0.008[13], 0.005[25], 0.002[26], 0.003[35], 0.001[36]
3rd measurement/mid-luteal	8.00 (3.00)	7.00 (2.00)	6.00 (3.00)	8.50 (2.00)	9.00 (2.00)	9.00 (1.00)	0.001	0.029[24], 0.001[25], <0.001[26], 0.036[34], 0.002[35], 0.001[36]
4th measurement/late luteal					9.00 (2.00)	9.00 (2.00)	0.880	Na
**Overall P value**	0.345	0.933	0.123	0.472	0.908	0.860		

*for 2mcW group the ordering of the data does not follow the actual timeline of measurement sessions (like for all other groups), but instead the phases of menstrual cycle. 1st measurement denotes mid-follicular phase, 2nd is ovulation, 3rd is mid-luteal and 4th measurement is late luteal phase.

Overall P value for between group comparisons was obtained with Kruskall-Wallis test. Post-hoc P values for between groups comparison were obtained with Mann-Whitney test: [1]Men (M); [2]Postmenopausal women (pmW); [3]Oral contraceptive users (ocW); [4]Women with an anovulatory cycle (aoW); [5]Women measured during four consecutive phases of one complete menstrual cycle, starting with mid-follicular phase (1mcW); [6]Women measured in four phases of two different, consecutive menstrual cycles (2mcW).

Overall P value for within group comparisons was obtained with Friedman test. Post-hoc P values for within group pairwise comparison were obtained with Wilcoxon Signed Rank test: [1]first measurement or mid-follicular phase; [2]second measurement or ovulation; [3]third measurement or mid-luteal phase; [4]fourth measurement or late luteal phase. Na – not applicable.
